# Differential circadian and light-driven rhythmicity of clock gene expression and behaviour in the turbot, *Scophthalmus maximus*

**DOI:** 10.1371/journal.pone.0219153

**Published:** 2019-07-05

**Authors:** Rosa M. Ceinos, Mauro Chivite, Marcos A. López-Patiño, Fatemeh Naderi, José L. Soengas, Nicholas S. Foulkes, Jesús M. Míguez

**Affiliations:** 1 Laboratorio de Fisioloxía Animal, Departamento de Bioloxía Funcional e Ciencias da Saúde, Facultade de Bioloxía and Centro de Investigación Mariña, Universidade de Vigo, Vigo (Pontevedra), Spain; 2 Institute of Toxicology and Genetics, Karlsruhe Institute of Technology, Eggenstein-Leopoldshafen, Germany; University of Lübeck, GERMANY

## Abstract

In fish, the circadian clock represents a key regulator of many aspects of biology and is controlled by combinations of abiotic and biotic factors. These environmental factors are frequently manipulated in fish farms as part of strategies designed to maximize productivity. The flatfish turbot, *Scophthalmus maximus*, represents one of the most important species within the aquaculture sector in Asia and Europe. Despite the strategic importance of this species, the function and regulation of the turbot circadian system remains poorly understood. Here, we have characterized the core circadian clock genes, *clock1*, *per1*, *per2* and *cry1* in turbot and have studied their daily expression in various tissues under a range of lighting conditions and feeding regimes. We have also explored the influence of light and feeding time on locomotor activity. Rhythmic expression of the four core clock genes was observed in all tissues studied under light dark (LD) cycle conditions. Rhythmicity of clock gene expression persisted upon transfer to artificial free running, constant conditions confirming their endogenous circadian clock control. Furthermore, turbot showed daily cycles of locomotor activity and food anticipatory activity (FAA) under LD and scheduled-feeding, with the activity phase as well as FAA coinciding with and being dependent upon exposure to light. Thus, while FAA was absent under constant dark (DD) conditions, it was still detected in constant light (LL). In contrast, general locomotor activity was arrhythmic in both constant darkness and constant light, pointing to a major contribution of light, in concert with the circadian clock, in timing locomotor activity in this species. Our data represents an important contribution to our understanding of the circadian timing system in the turbot and thereby the optimization of rearing protocols and the improvement of the well-being of turbot within fish farming environments.

## Introduction

Teleosts represent the largest and most diverse vertebrate group, in part due to their evolution in diverse habitats ranging from rivers, lakes and marshes to oceans. Furthermore, fish are economically extremely important. Aquaculture continues to expand worldwide, mainly because of over-fishing in the oceans that has led to many species being threatened. A major, on-going challenge for this industrial sector is to optimize both abiotic and biotic factors for each cultured fish species in order to maximize productivity.

In fish, the circadian clock plays a key role in the temporal organization of a broad range of biological processes and thereby plays a central role in optimizing survival. Light, temperature and food availability represent major environmental factors, indicative of the time of day (so-called *zeitgebers*), that serve to reset the circadian clock on a daily basis so that it remains synchronized with the 24 hours, day–night cycle. A key issue is how combinations of these environmental factors are integrated to regulate the circadian timing system. This integration step in large part appears to be species dependent and indeed represents one facet of adaptation to life in a particular ecological niche, e.g. catfish has a preference for eating during the dark phase, while in trout, feeding occurs during the light phase [1]. Desynchronization of the circadian system caused by artificially-induced changes in the relative timing of cycling environmental conditions may contribute to stress in animals thereby leading, for example, to a greater susceptibility to disease, a serious obstacle to aquaculture productivity.

At the molecular level the circadian timing system is based on a set of clock genes and proteins which are expressed in most cell types and are organized in translational-transcriptional feedback loops. In the mammalian clock at its simplest level, the transcription factors CLOCK and BMAL1 dimerize and active transcription of *per* and *cry* genes through their regulation of E-Box enhancer elements. When PER and CRY protein levels rise above a certain threshold in the cytoplasm, these two proteins are translocated to the nucleus where they inhibit CLOCK and BMAL1 activity [2]. Orthologs of the mammalian core circadian clock genes which are rhythmically expressed in most tissues have now been described in many fish species [3–9]. Although there are many similarities between the molecular elements of the mammalian and fish circadian timing systems, there also important differences in terms of the anatomical organization of the circadian system between these groups and even between teleost species. Thus, in mammals a central pacemaker localized in the suprachiasmatic nucleus (SCN) of the hypothalamus is regulated indirectly by light perceived via retinal photoreceptors. The SCN master clock governs the phases of peripheral tissue clocks harboured in most body cells. In contrast, in the case of fish, no direct counterpart of the single SCN clock has been identified. Instead, the retina and pineal gland of fish, both tissues with intrinsic photoreceptive mechanisms, harbour circadian oscillators and transduce lighting information into a circadian rhythm of melatonin production [10]. In addition, fish peripheral clocks are synchronized by direct light stimulation demonstrating the existence of photoreceptors and phototransduction cascades in most cells and tissues [6,11,12] and pointing to a significant degree of decentralization of the fish circadian timing system. Light and feeding time both serve as potent entraining signals for the circadian clock in fish, leading to the prediction of the existence of separate light and food entrainable oscillators (LEO and FEO respectively). However, the anatomical localization of these two oscillators and how they interact with each other, remains completely unknown. Downstream of this core timing mechanism, circadian rhythms of swimming activity have been widely documented in various fish species, including preprandial activity in anticipation of a regular feeding time (also known as food anticipatory activity (FAA) [13,14].

Flatfish such as Flounders and Plaice are a group of economically extremely valuable fish species with in particular, the turbot (*Scophthalmus maximus*) and Senegal sole (*Solea senegalensis*) representing major aquaculture species both in Europe and Asia. Studies of *S*. *senegalensis* have reported that it is a nocturnal species [15], with robust rhythms of melatonin and cortisol secretion,[15,16], as well as rhythmic clock gene expression in many regions of the brain and the liver [4,17]. On the contrary, the circadian system of turbot is less well studied. Daily variation in the levels of circulating melatonin have been reported in turbot and also newly metamorphosed larvae have been shown to be nocturnal while juveniles are diurnal [18,19]. The latter observations might be associated with the fact that flatfish use distinc spawning and nursery areas, so they need to adapt to the new environment. The circadian system clearly plays an important role in adaptation to changing environmental conditions. Interestingly, while turbot is an active visual predator, the Senegal sole is a non-visual predator [20,21] suggesting that light, the main synchronizer of the circadian system, might also be relevant for shaping the turbot's trophic activity.

Here, we wished to explore the function and regulation of the turbot's circadian timing system at the molecular and behavioral levels with the long term goal of contributing to the optimization of its aquaculture. We initially cloned partial sequences of four canonical components of the main transcription-translation feedback loop *clock1*, *per1*, *per2* and *cry1*. We then studied the expression of these genes in the eye, hypothalamus, gut, liver and muscle together with quantifying levels of behaviour under a LD cycle and a schedule feeding regime. We also examined the effect of transfer to constant darkness or constant light with regular, daily feeding or random feeding to explore the consequence of a lack of zeitgebers for circadian rhythmicity at the molecular and behavioural levels.

## Materials and methods

### Animals and experimental design

The turbot used in this study were provided by the Marine Research Centre (ECIMAT) of Vigo’s University (Spain). The experiments complied with the Guidelines of the European Union Council (2010/63/UE) and of the Spanish Government (RD 55/2013) for the use of animals in research. The Ethics Committee of the University of Vigo approved the procedures. *Scophthalmus maximus* were raised in an open circuit of sea water in 500L tanks, at 18°C, under natural photoperiod, and fed 4 times per day with a commercial pellet diet composed of 56% protein, 38% lipids and 6% carbohydrate (Skretting R-Europa 22) at the ECIMAT facilities. Two types of experiments were carried out with the following design.

#### Daily variation of gene expression

6 months old turbots with an average body weight of 95± 25g were acclimatized for 9 days in grey tanks of 100L (n = 10 fish per tank) in a 12:12-h light-dark cycle with lights on at 7:00 am. The light source was a 36W cool white fluorescent tube (Sylvannia F36watt/33-640 t8). The intensity of the light measured on the surface of the tanks was 130 lux. Three groups of fish were established, (i) (LD) was fed once per day with 1.5% of fish body weight at zeitgeber time (ZT) 2 (where ZT = 0 is defined as lights on), and sampled at ZT 3, 6, 9, 15, 18, 21 and 27 on the ninth day of acclimatization; (ii) (DD) fish were initially maintained in LD conditions with regular feeding but were then transferred to constant darkness on the ninth day with the same feeding schedule (feeding at Circadian Time (CT) 2) as the first group and sampled at CT 3, 6, 9, 15, 18, 21 and 27 and (iii) (R DD) fish were fed randomly during the light phase of an LD cycle, and then transferred to DD conditions with random feeding time before sampling under constant darkness at CT 3, 6, 9, 15, 18, 21 and 27. Eye, hypothalamus, anterior gut, liver and fast skeletal muscle from the anterior dorsal side were taken from 9 fish of each group and time point. Sampling in darkness was performed using a very weak dim red light directed away from the working area. The fish were anesthetized with 0.01% of phenoxyethanol and sacrificed by decapitation. Tissues were stored at -80°C until subsequent analyses.

#### Daily locomotor activity

In order to investigate turbot circadian behaviour, animals were relocated in opaque, grey aquarium tanks of 30L, with dimensions of 60x20x45cm (length/height/width) containing a 14cm column of sea water at 18°C and constant air flow. 18 turbots were divided into two groups. One group was fed regularly at ZT2 or CT2 and a second group was fed at random times during the light phase or subjective light phase. Both groups were subjected to different lighting regimes in three independent experiments. Experiment 1: 11 days exposure to 24 hours LD cycles (12h light and 12h darkness) followed by 3 days of constant darkness and finally 19 days of LD. Experiment 2: 9 days of LD, plus 4 days of constant light (LL) and 5 days of LD. Experiment 3: 8 days of cycles of 8h of light and 16h of darkness (8L:16D). During experiment 3 animals were fed at ZT23 (at the end of dark phase).The intensity of light measured at the top of the tanks was 380 lux. At the beginning of the experiment, fish were juveniles of 3 months old with a body weight close to 16g.

### Cloning of core clock genes from turbot

The amplification of partial cDNA sequences from total RNA extracts of the hypothalamus of the *period 1* (*per1*), *cryptochrome* 1 (*cry1*), *circadian locomotor output cycles protein kaput* (*clock 1*) and *period* 2 (*per2*) genes was performed using RT-PCR with primers designed using sequence contigs from the turbot genome database [22,23]. The PCR target sequences for the four genes were selected by blasting clock gene sequences derived from several species of teleost, (mainly *Salmon salar*) against a turbot genome database https://www.ncbi.nlm.nih.gov/genome?term=Scophthalmus[orgn]%20OR%20Scophthalmus%20maximus[orgn]. PCR primers were designed to target conserved regions of teleost fish homologs identified by multiple sequence alignment using Clustal Omega software (https://www.ebi.ac.uk/Tools/msa/clustalo/). All primers were designed using Primer 3 (http://bioinfo.ut.ee/primer3-0.4.0/). All PCRs were run on a Peltier Thermal cycler by denaturing at 94°C for 5 min followed by 40 cycles of amplification at 94°C for 30 s, 54–60°C depending of the primers, for 30 s and 72°C for 1 min. The PCR reaction was carried out using DreamTaq Green PCR Master Mix (Promega). The PCR products of the expected size were electrophoresed on a 1% agarose gel, viewed under UV light and then purified, cloned into the pGEM Teasy vector (Promega), transformed with JM109 competent cells and sequenced on both strands with SP6 and T7 promoter-specific primers. The sequences were compared with the GenBank database by using the BLAST algorithm https://blast.ncbi.nlm.nih.gov/Blast.cgi. Coding sequences were deposited in GenBank and accession numbers are listed alongside cloning primers in supplementary S1 Table. During the preparation of this manuscript, the annotation and complete sequence of proteins encoded by genes from the *S*. *maximus* genome were made available on Genbank (S2 Fig).

### Analysis of mRNA expression

Total RNA was extracted using Trizol Reagent (Ambion) from hypothalamus, eye, gut, muscle and liver following the manufacturer’s protocol. RNA pellet was diluted in RNAse-free water and the quality and quantity was measured using Nanovue plus. Contaminating genomic DNA was removed using RQ1 DNase (Promega). The first strand cDNA was obtained by using M-MLV reverse transcriptase and random primers (Promega) following the manufacturer’s protocol. For real-time quantitative RT-PCR analyses the specific primers used are listed in supplementary S1 Table. Each real-time qPCR was performed using Universal Sybr Green qPCR master mix (Thermo scientific). The reaction was carried out in duplicates using a MyIQ Bio-Rad device and the thermal cycling profile consisted of an initial denaturation at 95°C for 10 min, followed by 40 cycles of denaturation at 95°C for 15 s and annealing/extension at 60°C for 30 s. Water was used as the negative control for the qPCR analysis. Product specificity was confirmed by melting curve analysis while the quality of the designed primers for qPCR was assessed based on the gradient and efficiency of primer calibration curves using cDNA from hypothalamus. Normalized relative mRNA abundance was calculated using the comparative delta-CT method [24] with β-actin serving as a reference gene for normalized gene expression in hypothalamus, eye and muscle, ribosomal protein L8 (rpl8) for gut and the average of threshold cycles (CTs) β-actin and RNA polymerase II subunit D mRNA (rpsd) for liver.

### Activity data analysis

Locomotor activity was recorded using a system which counts the frequency with which an infrared light beam is interrupted by the passage of a fish. Each of two tanks was equipped with two photocells. One photocell was fixed 2.5cm above the bottom of the tank, on the front wall, and just in front of the position of an automatic feeder (which was suspended above). The second photocell was fixed 8cm above the bottom of the tank but on a side wall, so that its orientation was perpendicular to that of the first photocell, but still in a position close to the automatic feeder. Interruptions of the infrared beam were registered every 10 minutes. The data acquisition from the photocell was done using the software Micronec ADQ-D16-ICP (MICRONEC@micronec.com).

### Statistical analysis

Raw locomotor activity data were analysed and plotted using the chronobiological software El Temps, designed by Prof. Díez-Noguera (University of Barcelona). Data were displayed in a double-plotted actogram, periodogram or bar graph. The average of counts/10min, 2 hours preceding meal time, light and dark phases of 30 consecutive days in LD or 3 days in DD was determined using Excel software. One-way ANOVA was used to evaluate the effect of regular and random meal delivery on the distribution of locomotor activity between phases. The normalized gene expression data are shown as mean ± SEM. To ascertain whether changes in gene expression levels over a 24h sampling window show circadian rhythmicity, one-way ANOVA followed by Tukey’s test and cosinor analysis were performed. When data did not follow a normalized distribution, then the nonparametric Kruskal-Wallis H Test was applied. To determine the significance of changes in gene expression, between two different conditions, e.g. light-dark cycle vs constant darkness or regular feeding time vs random feeding time at every time of the day, two-way ANOVA followed by a Bonferroni test was performed. *p*<0.05 was considered as a statistically significant difference. Statistical analyses were performed using GraphPad Prism 5.0 and Excel software. Mesor, amplitude, and acrophase were estimated by cosine analysis according to the software CRONO designed by Prof. Díez-Noguera (University of Barcelona).

## Results

### Cloning of core circadian clock genes from turbot

We chose to use a RT-PCR approach to clone circadian clock gene homologs from turbot. Primers were designed to target conserved clock gene sequences identified by a Blast search of turbot genome sequence database [22,23]. Subsequently, four partial cDNA sequences were amplified using RT-PCR from turbot hypothalamus RNA namely, 798bp of *smperiod1* (*smper1*), 870bp of *smperiod2* (*smper2*), 851bp of *circadian locomotor output cycles protein kaput* (*smclock1*) and 964bp of *smcryptochrome1* (*smcry1*). The corresponding amino acid sequences were then used to perform a blast alignment with other teleost clock proteins. smPer1 showed a 79% of identity with the Per1 amino acid sequence from *P*. *olivaceus*; smPer2 showed 98% identity with Per2 from *P*. *olivaceus*; smClock showed 98% and 94% identity with Clock from *P*. *olivaceus* and *D*. *rerio* respectively and smCry1 exhibited 97% identity with Cry1 of *P*. *ovlivaceus*. Smart Blast indicated that smPer2 incorporated a conserved PER-ARNT-SIM (PAS) domain, smClock contained two PAS domains and smCry1 carried a DNA photolyase FAD binding domain. A phylogenetic tree illustrates the phylogenetic relationships of the turbot clock protein sequences with those of other vertebrate species including mammals, birds, amphibia, reptiles and fish (the species are listed in supporting information S2 Table). The four clock proteins were grouped close to their respective homologous proteins within clades constituted by fish species.

### Daily rhythms of clock gene expression in central and peripheral tissues

**Fig 1:**
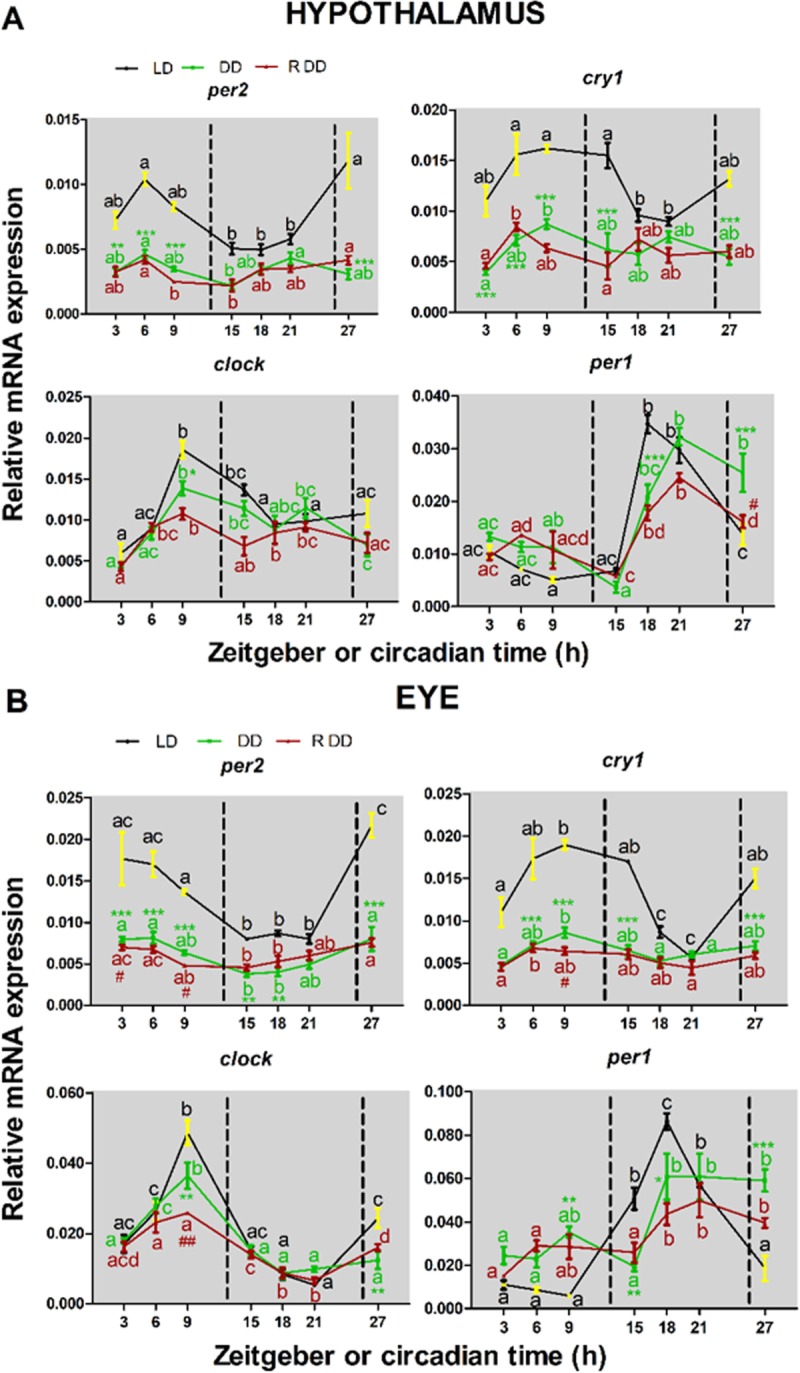
Daily expression of clock genes in central tissues from turbot. mRNA relative expression profiles of per2, cry1, clock and per1 in hypothalamus (A) and eye (B) of fish acclimatized to LD and regular feeding time at ZT2 (LD, black line), 24h in DD and regular feeding time at CT2 (DD, green line) and 24h DD and random feeding time during the light phase (R DD, red line) measured by qPCR. Data represent mean ± SEM of three (eye) or five animals per time point. The dotted black lines delimit the duration of the night in LD conditions or the duration of the subjective night in DD. Different letters of the same colour indicate statistical difference inside a group (p<0.05 One-way ANOVA followed by Tukey’s test). Asterisks indicate a statistical difference from LD conditions (*p<0.05, **p<0.01, ***p<0.001 Two-way ANOVA followed by Bonferroni’s test). Hash indicates statistical difference from regular feeding time (#p<0.05, ##p <0.01, ###p<0.001 Two-way ANOVA followed by Bonferroni’s test). These time points correspond to ZT 3, 6, 9, 15, 18, 21, 27h. ZT0 represents lights on and ZT12, lights off.

Circadian clock function relies upon the coordinated cycling of mRNA levels of core clock genes. Therefore, in order to characterize the turbot clock in more detail, we next explored the *smclock1*, *smper1*, *smper2* and *smcry1* temporal expression patterns in central (hypothalamus and eye) and peripheral tissues (gut, liver and muscle) of fish initially exposed to 12h light and 12h dark (LD) cycles being fed regularly at ZT2 and subsequently, for the final 24 hours sampling period, either maintained under LD conditions (with continued feeding at ZT2) or shifted to constant darkness (Fig 1, DD) (with feeding at Circadian time (CT) 2). In the hypothalamus and eye in LD (black traces, Fig 1A and 1B respectively), significant daily oscillations of the mRNA levels for all four clock genes were evident (for cosinor analysis, see Table 1). Furthermore, *smper2* and *smcry1* showed identical acrophase distributions in both tissues (Fig 1 and Table 1), (4.31h in the hypothalamus and 4.17h in the eye for *smper2*, and 9.5h in hypothalamus and 9.44h in the eye for *smcry1*). The rhythmic expression of *smclock1* and *smper1* displayed opposite phases with *smclock1* peaking at the end of the light phase, while *smper1* peaked during the middle of the dark phase. The time difference between the *smclock1* and *smper1* peaks was comparable in the hypothalamus (9.03h) and eye (9.29h) (Fig 1). In the hypothalamus of fish shifted to DD (green traces), mRNA levels of *smclock1* and *smper1*, still displayed significant rhythmicity (Cosinor Analysis, p<0.01 in both genes). However, although *smper2* and *smcry1* mRNA expression was arrhythmic in the hypothalamus as tested by cosinor analysis, one-way ANOVA did reveal a statistical significant time-of-day variation in expression for both genes F (6, 22) = 6.65; p<0.001 for *smper2*; F (6, 24) = 2.65; p< 0.05 for *smcry1*). Analysis of two-way ANOVA indicated that the interaction between lighting condition and time-of-day was significant for *smper2* F (6, 48) = 5.36; p = 0.0003 and *smper1* F (6, 48) = 7.84; p = 0.0001. Following transfer to DD, all four clock genes continued to exhibit rhythmic expression in the eye (two-way ANOVA revealed statistical significant interaction for *smper2* F (6, 42) = 7.00; p<0.0001); *smcry1* (F (6,37) = 11.95; p<0.0001); for *smclock1* F (6,41) = 4.17; p = 0.0023) and for *smper1* F (6,37) = 10.90; p<0.0001)).

**Table 1:** Rhythmic parameters of clock gene expression.

		LD FEEDING TIME AT ZT2				DD FEEDING TIME AT CT2				DD RANDOM FEEDING TIME				
	Tissue	Mesor (r.e.)	Amp. (r.e.)	Acrop (h)	Cosinor*p*	Mesor (r.e.)	Amp. (r.e.)	Acrop. (h)	Cosinor*p*	Mesor (r.e.)	Amp. (r.e.)	Acrop (h)	Cosinor*p*
*Per2*	HT	0.008	0.003	4.31	**<0.01**				n.s	0.003	0.001	0.35	**<0.01**
	Retina	0.013	0.006	4.17	**<0.01**	0.006	0.002	4.17	**<0.01**	0.006	0.002	1.4	**<0.01**
	Mus.	0.003	0.001	10.38	**<0.05**				n.s				n.s
	Gut	0.008	0.003	8.43	**<0.01**	0.005	0.003	5.34	**<0.01**	0.003	0.001	6.34	**<0.01**
	Liver				n.s				n.s				n.s
*Cry1*	HT	0.013	0.004	9.5	**<0.01**				n.s				n.s
	Retina	0.014	0.007	9.44	**<0.01**	0.007	0.002	9.49	**<0.01**	0.006	0.001	9.58	**<0.01**
	Mus.	0.003	0.002	11.08	**<0.01**	0.001	0.000	17.07	**<0.05**	0.000	0.000	17.04	**<0.05**
	Gut	0.009	0.007	11.05	**<0.01**				n.s	0.002	0.001	12.05	**<0.05**
	Liver	0.057	0.029	12.09	**<0.01**				n.s				n.s
*Clock1*	HT	0.012	0.005	11.37	**<0.01**	0.01	0.004	13.04	**<0.01**				n.s
	Retina	0.021	0.019	9.03	**<0.01**	0.018	0.014	9.16	**<0.01**	0.016	0.01	8.42	**<0.01**
	Mus.	0.005	0.002	13	**<0.01**	0.005	0.002	21.44	**<0.01**	0.003	0.001	16.23	**<0.05**
	Gut	0.008	0.01	10.53	**<0.01**	0.005	0.005	12.02	**<0.01**	0.004	0.004	12.29	**<0.01**
	Liver	0.02	0.012	11.37	**<0.01**	0.004	0.002	16.20	**<0.01**	0.007	0.003	14.11	**<0.01**
*Per1*	HT	0.015	0.014	20.39	**<0.01**	0.017	0.013	22.52	**<0.01**	0.014	0.007	22.13	**<0.01**
	Retina	0.037	0.035	18.32	**<0.01**	0.043	0.026	21.31	**<0.01**	0.033	0.01	20.32	**<0.01**
	Mus.	0.009	0.008	20	**<0.01**				n.s				n.s
	Gut	0.021	0.017	18.46	**<0.01**	0.025	0.016	23.48	**<0.01**	0.014	0.008	3.16	**<0.01**
	Liver	0.389	0.328	20.30	**<0.01**	0.182	0.07	2.5	**<0.01**	0.157	0.08	3.21	**<0.01**

Fish kept under: LD and feeding time at ZT2, 24h DD and feeding time at CT2 and 24h DD and random feeding time. Mesor and amplitude (Ampl.) are expressed in relative expression units (r.e) and acrophase (Acrop) in hour: minutes. Parameters are derived from cosinor analysis using Chrono., software developed by Prof. A. Díez Noguera.

**Fig 2:**
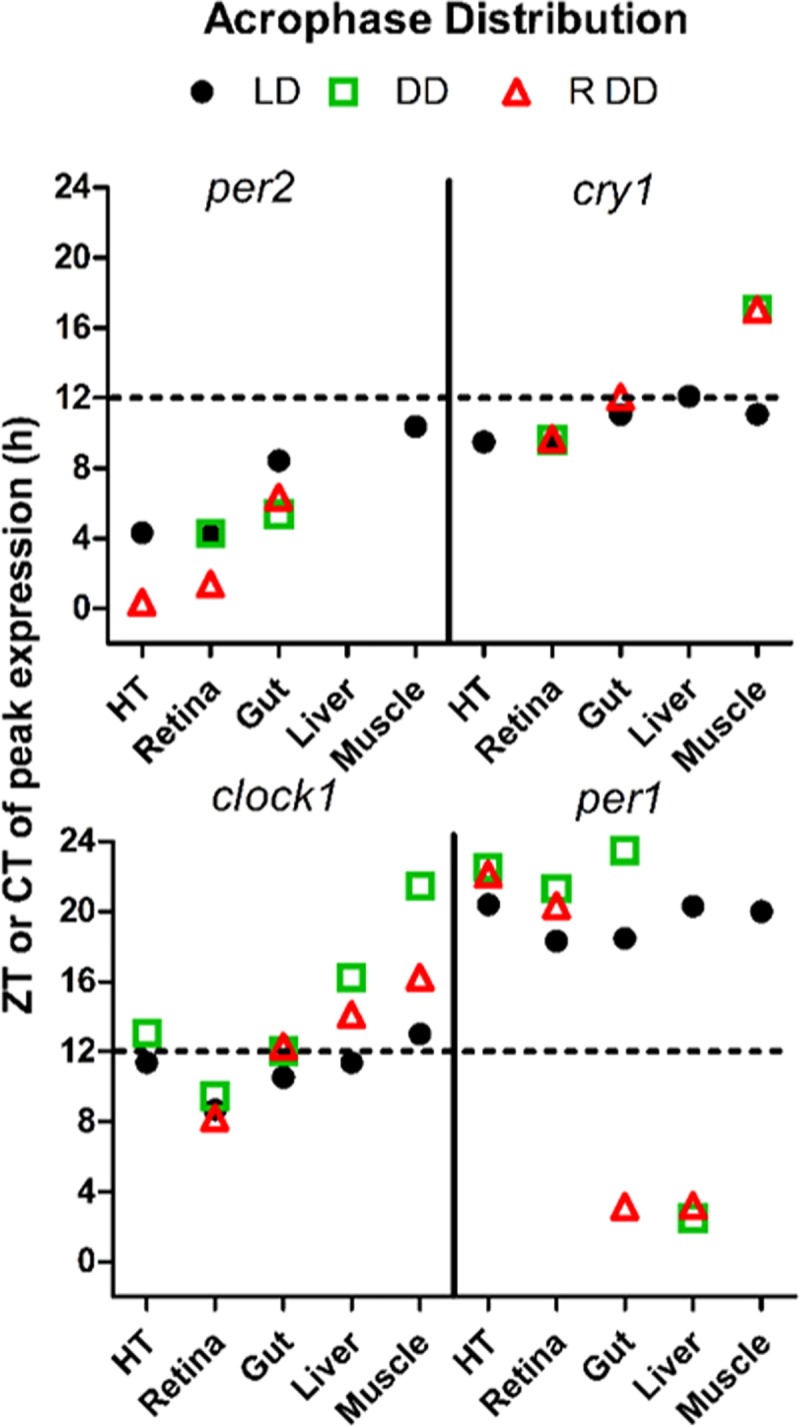
Acrophase distribution of per2, cry1, clock and per1 from turbot. Fish kept under schedule feeding time and LD (black points) or DD (green square) and under free-running conditions (red triangle). Only the acrophases of the genes that showed significant rhythms as confirmed by cosinor analysis were represented. The cry1 acrophase was shifted under DD in muscle, meanwhile the per1 acrophase shifted under free-running conditions in gut and under DD and schedule feeding and free running conditions in liver. The dashed line on the Y axis shows the boundary between the light phase and the dark phase. ZT0 is defined as the time when lights are switched on.

**Fig 3:**
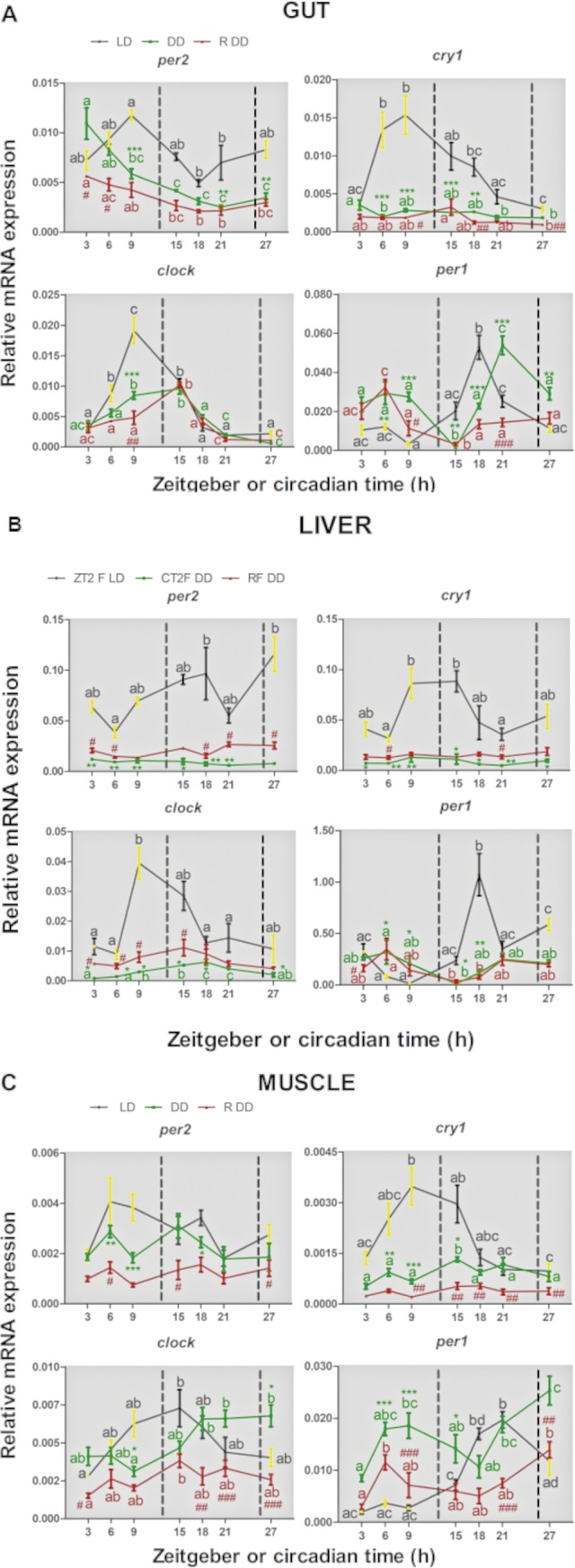
Daily expression of clock genes in peripheral tissues from turbot. mRNA relative expression profiles of per2, cry1, clock and per1 in gut (A), liver (B) and muscle (C) of fish adapted to LD and regular feeding time at ZT2 (LD, black line), 24h DD and regular feeding time at CT2 (DD, green line) and 24h in DD and random feeding time during the light phase (R DD, red line) measured by qPCR. Data represent mean ± SEM of five animals per time point. The dot black lines delimit the duration of the night in LD or the duration of the subjective night in DD. Plots and statistical analysis results are indicated as described for Fig 1.

In peripheral tissues under LD conditions (Fig 3, black traces), the four canonical genes displayed significant rhythmic expression in the gut (A), liver (B) and muscle (C) with the only exception being the expression of *smper2* in the liver, which was arrhythmic. The acrophase distribution for *smper2* (8-10h), *smcry1* (11-12h), *smclock1* (11-13h) and *smper1* (18-20h) were very similar among the gut, liver and muscle (Fig 2, black points), pointing to the circadian clocks in these tissues, which all share important roles in metabolism, being synchronized. Furthermore, the timing of the nocturnal peak of *smper1* expression observed in all the peripheral tissues was very similar to that in central tissues (Table 1 and Fig 2).

Upon transfer to constant darkness, the expression of *smper2* in the muscle and of *smcry1* in the gut and liver became arrhythmic (Fig 3A, 3B and 3C, green traces). However, in the case of *smclock1*, rhythmicity was detected in all tissues under DD conditions, although the acrophase of *clock* rhythmic expression was delayed by 5 and 8 hours in liver and muscle, respectively (Fig 2 and Table 1). Instead, *smper1* maintained rhythmic expression in the gut and liver upon transfer to DD but not in muscle. Comparing clock gene expression under LD and DD conditions, we observed gene and tissue specific changes in the basal levels of expression. Thus, in the gut, the expression levels of *smper2* were significantly lower at CT 9, 21 and 27 compared to those at the same times under LD (two-way ANOVA F = 11.07; p<0.0001), while *smcry1* expression levels were reduced throughout the entire 24 hours period (two-way ANOVA F = 10.06; p<0.0001). *smclock1* expression levels were significantly lower in DD at CT9 compared with ZT9 with consequently a lower amplitude rhythm. For rhythmic *smper1* expression, the peak under DD was shifted by 5h to the end of the subjective night, although it was the only gene without changes in the basal levels of expression. In the liver, basal levels of all four clock gene transcripts were significantly lower in DD compared to LD. Hepatic *smper1* showed a phase shift in the light phase and its mesor was reduced by 53%. In the muscle, *smcry1* showed a low-amplitude circadian oscillation.

### Circadian rhythms of clock gene expression in central and peripheral tissues

The fish analysed under DD conditions were still exposed to a zeitgeber, namely a regular feeding regime. Therefore, in order to explore circadian clock function in turbot under free running conditions, we next tested for the persistence of entrained clock gene expression rhythms upon transfer to constant darkness but with a randomized feeding time regime. Fish were entrained to 10 days of LD cycles and fed once a day at random times during the light phase. Subsequently, they were sacrificed under constant darkness at 3-6h intervals during a 24 hours sampling window, again with a random feeding time. In central tissues, *smcry1* and *smclock1* expression was arrhythmic in the hypothalamus (Fig 1A RDD, red trace and Table 1), whereas all four genes displayed rhythmic expression in the eye (for cosinor analysis see Table 1) (Fig 1B RDD, red trace and Table 1). In peripheral tissues, the expression of all four clock genes was down regulated compared with the LD adapted samples (Fig 3A, 3B and 3C, RDD red traces). Furthermore, *smper2* and *smper1* expression was arrhythmic in the muscle and *smcry1* showed arrhythmic expression in the liver (Table 1). Comparing the schedule feeding with the random feeding fed fish in DD conditions, in cases where rhythmic clock gene expression persisted, the acrophase distributions remained similar in all tissues with the exception being *smper1*, where it was shifted from the subjective night to the subjective day in the gut and liver, and *smcry1* in the muscle, where it was shifted from the subjective day to the subjective night (Fig 2).

### Coupling of swimming activity and FAA to the light phase

**Fig 4:**
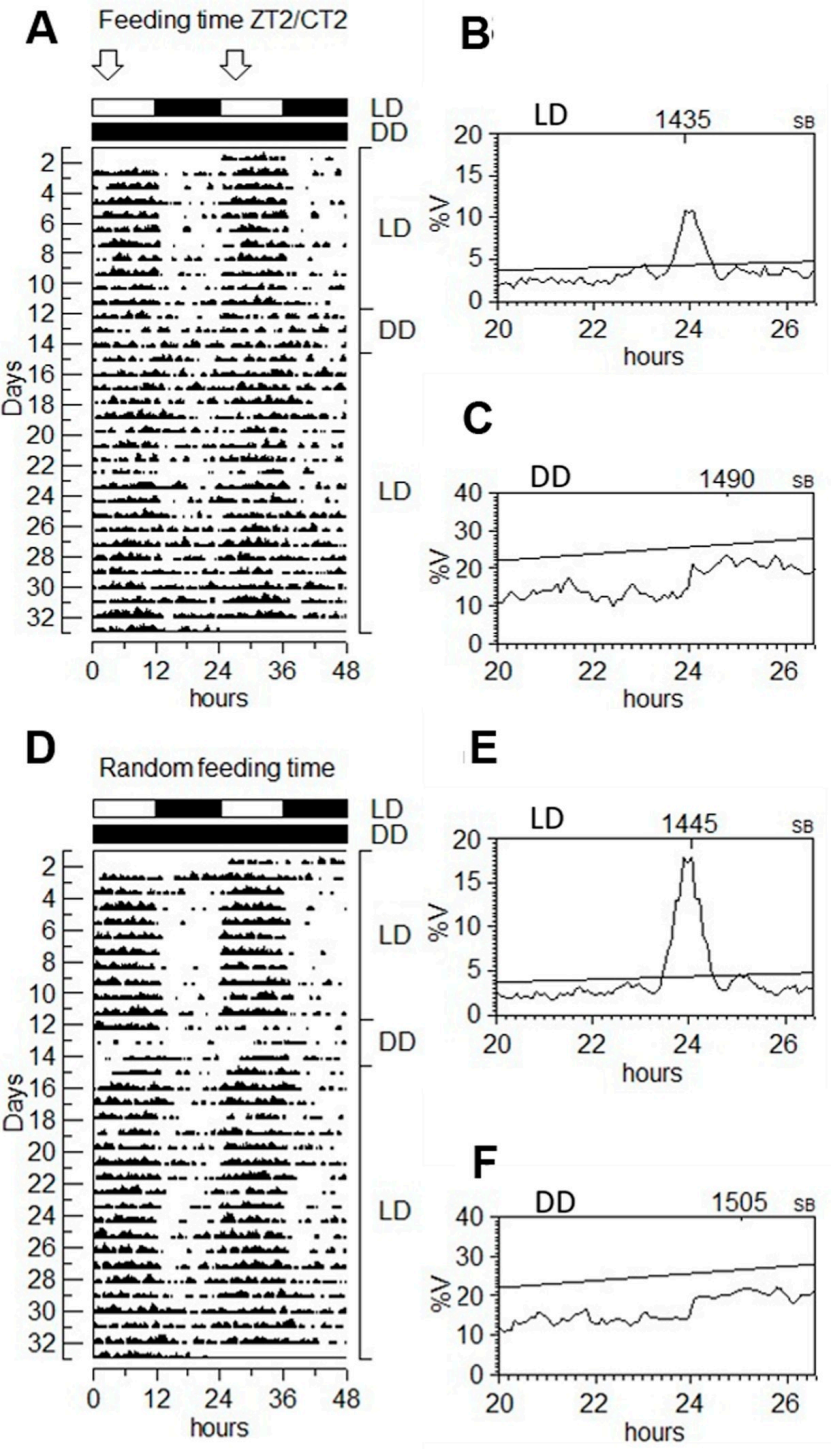
Diurnal locomotor activity of turbot in LD-DD-LD lighting conditions. A, D) Representative actograms of turbot swimming activity. Recording activity during 33 days is represented by double plot of two consecutive days. The white and black bars above the actograms indicate light and dark periods. The arrows above the white bar indicate the time where food is delivered. Fish were kept in LD and fed at ZT2 for 33 days (A), or randomly fed during the light phase (D). From day 12 to 14 fish were in DD and then returned to LD. Fishes showed daily periodicity in LD (B, E), but not in DD (C, D). Turbot displayed its activity mainly during the light phase.

**Fig 5:**
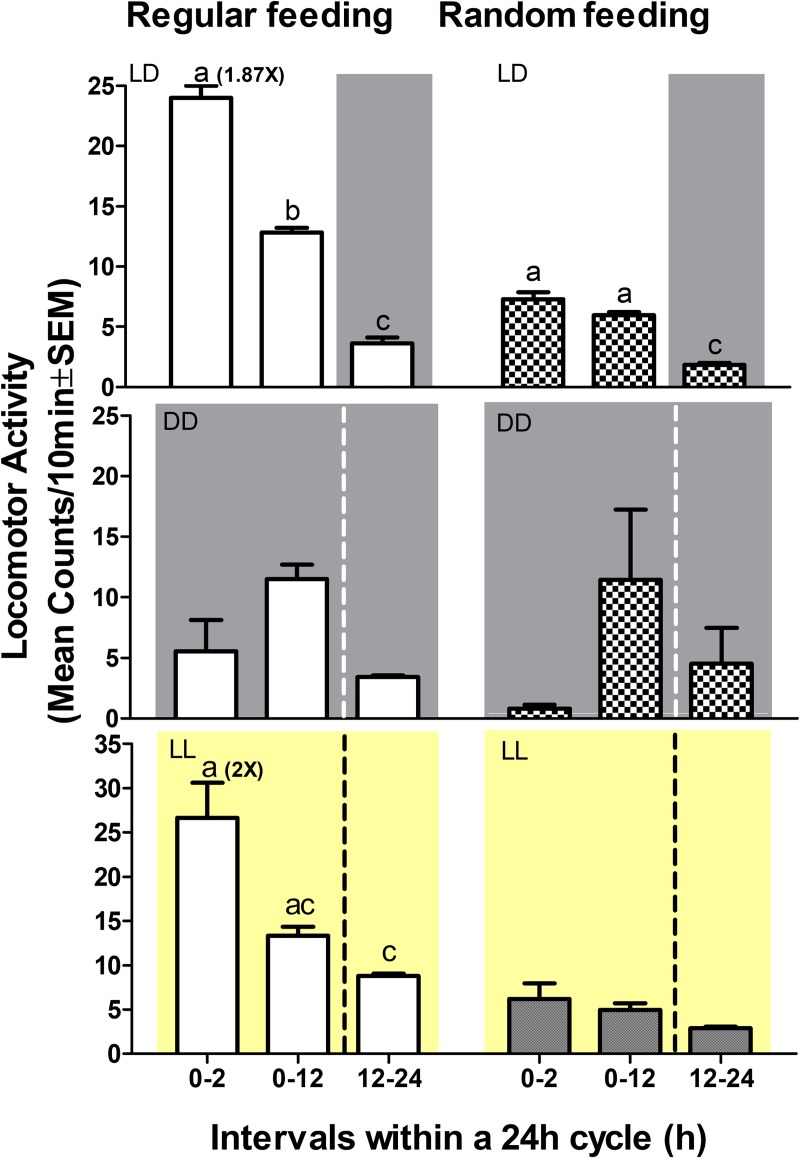
**Preprandial locomotor activity in LD (top), DD (mid) and LL (bottom).** Activity levels during the photophase (0–12) and scotophase (12–24) of a 24-h cycle from turbot. Within the photophase, activity levels were analyzed 2h before lunch time (0–2) and during the entire light phase (0–12) either in the group with a schedule feeding time at ZT2 or in the group randomly fed, in LD (top), DD (mid) and LL (bottom). Bar graphs show mean ± SEM. Significant differences between groups are indicated by letters. Scheduled feeding significantly stimulates increases in locomotor activity before feeding time under LD and LL but not under DD conditions (Kruskal-Wallis one way anova: H = 73.73, df 3, p<0.05 and post hoc by the Dunn’s test p<0.001). No changes in locomotor activity were observed upon the onset of light period compared with the whole light phase in randomly fed animals.

Previous reports of diurnal and nocturnal activity patterns for juvenile turbot [18,19] lead us to explore whether the juvenile turbot used in our study show daily rhythms of activity and also the influence of the feeding schedule on their behaviour. We therefore exposed two groups of fish to LD cycles, one being fed once a day at ZT2 while the second group was fed at random times. After more than 30 days of entrainment, the fish were transferred for three days to constant darkness and then the LD cycle was re-established with a continuation of the feeding regime. Actograms and periodogram analysis of locomotor activity under scheduled and random feeding in LD conditions (Fig 4A, 4B, 4D and 4E) showed a rhythmic, predominantly diurnal pattern of activity. In contrast, fish activity was arrhythmic during the three days in DD regardless of whether food was regularly or randomly provided. Under LD conditions, in the schedule feeding group the mean levels of activity preceding regular mealtime (23±1.15) was significantly higher (1.87 fold increase) than the total level of activity (13±0.13) observed for the light phase (Dunn’s Multiple Comparison test, p<0.001), (Fig 5, top-left), indicative of food anticipatory activity (FAA). In addition, following the administration of food there was a gradual return to basal levels of activity. In contrast, in the randomly fed group, immediately before (7±0.57) and after (6±0.27) meal time, activity was comparable (Fig 5, top-right). Furthermore, under DD conditions, meal time did not have a significant effect on locomotor activity for either the scheduled feeding or randomly fed groups before the arrival of food, (Fig 5, middle).

**Fig 6:**
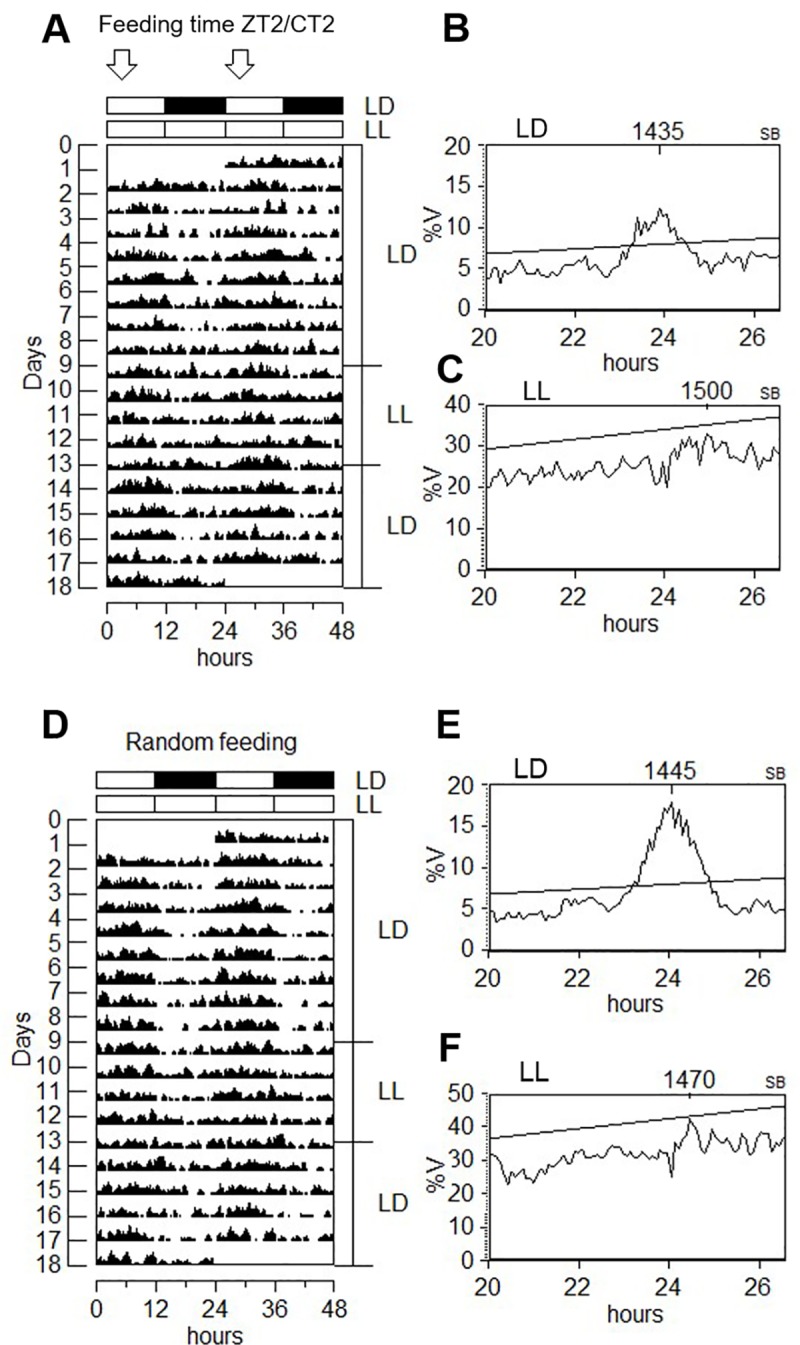
Diurnal locomotor activity from turbot in LD-LL-LD lighting conditions. Recording activity during 18 days is represented by double plot of two consecutive days and represented by actograms (A, D). The white and black bars above the actograms indicate light and dark periods. The arrows above the white bar indicate the time where food is delivered. Fish were kept in LD and fed at ZT2 for 8 days (A), or randomly fed during the light phase (D). From day 9 to 12 fish were in constant light (LL) and then returned to LD. Fish showed daily rhythmicity in activity under LD conditions (B, E), but they were arrhythmic in LL (C, F). Turbot displayed activity during the light phase in LD.

**Fig 7:**
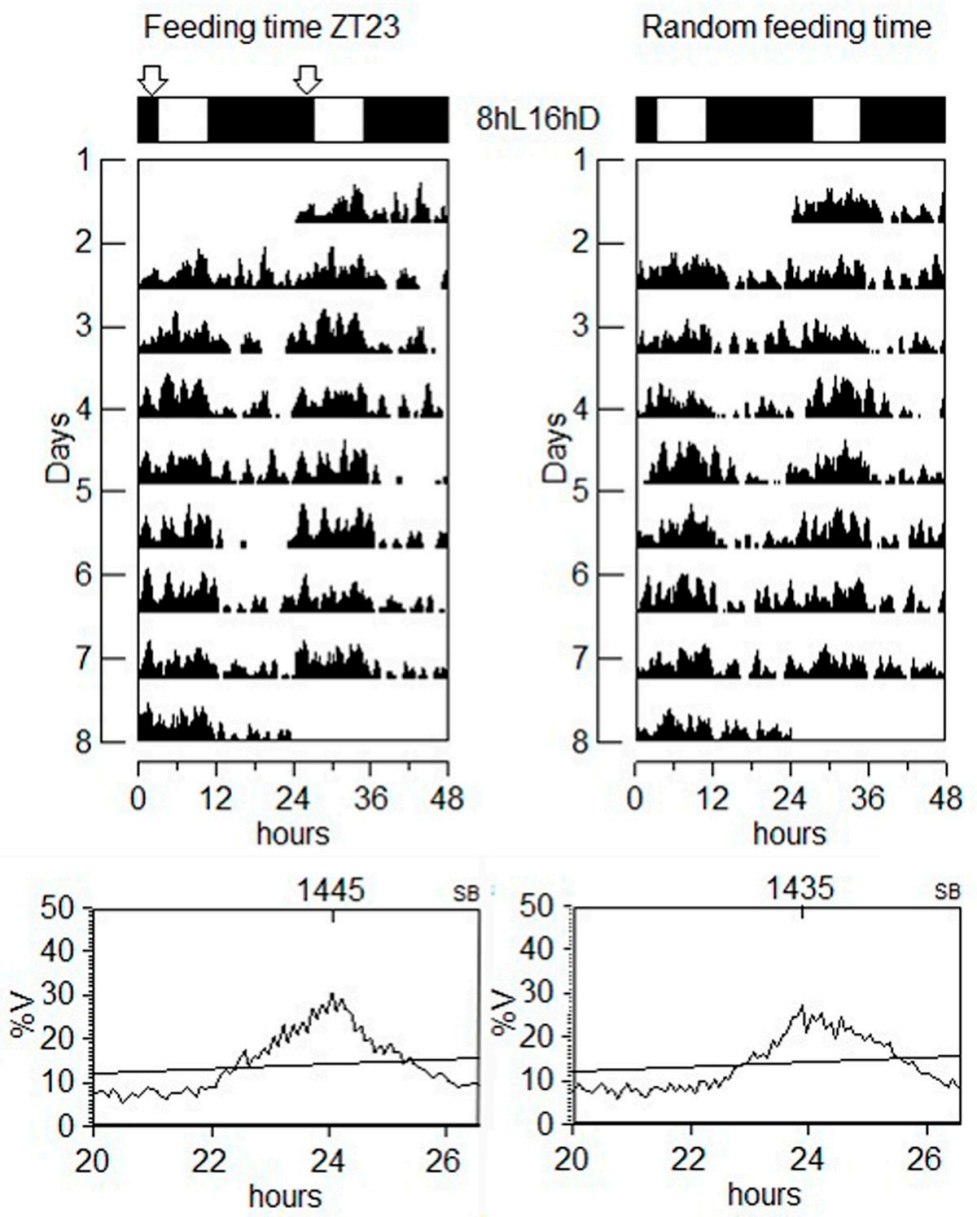
Diurnal locomotor activity from turbot under short photoperiod (SP). Recording activity during 8 days is represented by double plot of two consecutive days and represented by actograms. The white and black bars above the actograms indicate light and dark periods. The arrows above the white bar indicate the time where food is delivered. Fish were kept in SP and fed at the end of the scotophase (ZT23) (left), or randomly fed during the light phase (right). Fish showed rhythmic activity in SP.

**Fig 8:**
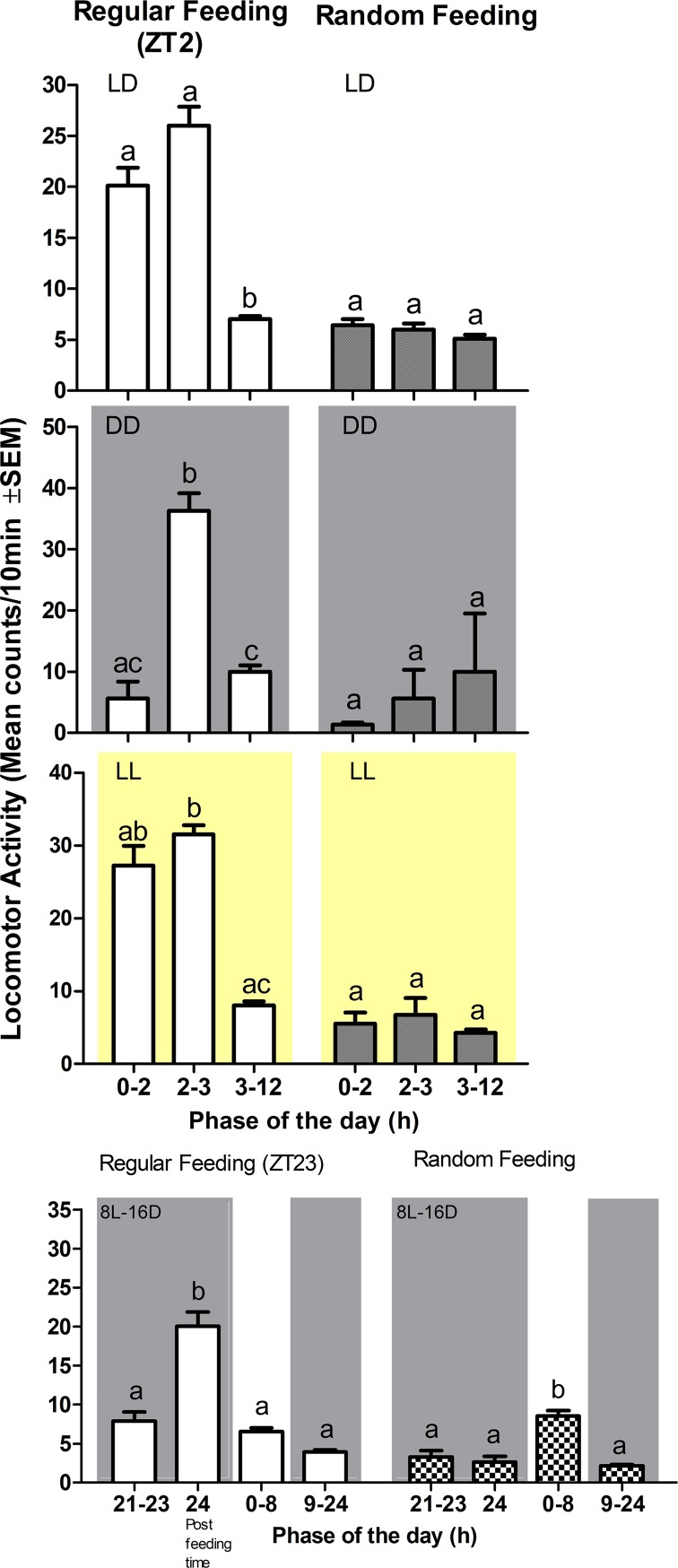
Feeding behaviour in fish under four different lighting regimes. One group of animals were entrained to eat at ZT2 under LD, at CT2 under DD or LL, and at ZT23 under short photoperiod (left) and a second group was fed at random times (right) during the scotophase or subjective scotophase. Bars represent activity levels: 2h before the arrival of food (0–2) under LD, DD or LL and 1h before the arrival of food under short photoperiod; when meal is provided (2–3) under LD, DD or LL and (24) during short photoperiod; during the rest of the day (3–12) under LD or subjective day under DD or LL, and during the entire light phase (0–8) and during the entire dark phase (9–24) under short photoperiod. Turbot failed to show food searching behavior when food was supplied regularly in the dark phase. Instead animals were very active when food was presented, independently of the light regime. (Kruskal-Wallis one way anova: H = 18.92 LD; H = 6.16 DD; H = 8.11 LL; H = 25.42, df 4, p<0.0001 and post hoc by the Dunn’s test p<0.05).

We next questioned whether the FAA behavior that precedes mealtime in LD conditions might be light-dependent. We exposed juvenile turbot to constant illumination together with regular feeding or a random feeding time (Fig 6). The animals were arrhythmic in LL with or without regular feeding (Fig 6C and 6F), however, they retained the preprandial increase in activity under a regular feeding schedule (Fig 5 bottom). In order to verify the absence of FAA under darkness and to avoid changing the feeding schedule to which fish had been acclimatized for about two months, the animals were adapted to a short photoperiod of 8 hours of light and 16 hours of darkness. Under these conditions the extended night period then incorporated the regular feeding time at ZT23 and the animals showed daily locomotor activity variations matching the photoperiod (Fig 7), but with no increases in activity prior to the arrival of food (Fig 8, 8L-16D bottom). In addition, an increase in activity was also observed immediately after the administration of food under all lighting regimes, possibly the result of fish reacting acutely to the smell of food in the water, or to the vibrations caused by adding the food to the water (Fig 8, LD, DD, LL, 8L-16D).

## Discussion

Our work provides a first glimpse of the organization of the circadian timing system of the turbot, *Scophthalmus maximus*, a highly relevant flatfish species for the aquaculture industry throughout Asia and Europe. We report the cloning of partial sequences of core clock genes representative of the positive and negative elements of the core transcriptional-translational feedback loop, namely *clock1* and *per1*, *per2* and *cry1*, respectively. The four genes displayed rhythmic expression in both central (retina and hypothalamus) and peripheral (gut, muscle and liver) tissues. Light cycles and feeding time were able to entrain rhythmic clock gene expression, although, while light exerted a generalized effect on all oscillators, the effects of feeding were more evident in the intestine and liver. Under free-running conditions, the rhythms of expression of clock gene expression persist confirming the presence of a self-sustaining circadian oscillator. On the contrary, circadian rhythmicity in the turbot's locomotor activity as well as FAA depends on the continued exposure to a light /dark cycle and light, respectively, thereby implying a strong coupling of locomotor and foraging activities with the light phase.

### Regulation of clock gene expression in turbot

We reveal that the expression of all four clock genes, *smper2*, *smcry1*, *smclock1* and *smper1*, showed daily variations in all central (hypothalamus and retina) and peripheral tissues (gut and muscle), except for *smper2* that was arrhythmically expressed in the liver. These results indicate that multiple oscillators are widely distributed throughout the turbot body, closely resembling the situation in other vertebrates, including other teleost species e.g. *Carassius auratus*, *Danio rerio* [7,11], *Oncorhynchus mykiss* [25], *Sparus autrata*, *Solea senegalensis* and *Paralichthys olivaceus* [9,17,26]. The rhythmic expression pattern of genes in the mammalian clock encoding negative elements of the core clock machinery (per and cry) tends to be characteristically phase shifted with respect to those encoding positive elements (Clock and Bmal). In turbot, the expression profile of *smclock*, *smper1* and *smper2* matches this general trend. Thus, the acrophase of *smclock* coincides with the end of the light phase while the acrophase of *smper1* and *smper2* occurs at the end of the dark phase / onset of the light phase. Interestingly the expression pattern of the *smcry1* gene matches more closely that of *smclock1* (Figs 1 and 2). However, information concerning the levels of clock proteins will be required to assess the functional significance of these phase differences.

Despite the evidence for the functionality of the turbot circadian system observed in this study, turbot locomotor activity is immediately arrhythmic upon transfer to DD. Consistent with this observation, in muscle, a tissue directly involved in locomotion, *smper2* and *smper1* genes, negative effectors within the clock machinery, were arrhythmic in DD regardless of feeding time. These findings point to the importance of light for shaping clock function and clock outputs such as locomotor activity. In zebrafish cells, the expression of the *per2* and *cry1* genes is controlled by the clock, as well as being acutely induced upon direct light exposure of a range of tissues and cell types [27–29]. A light-responsive module has been described in the promoter of zebrafish *per2*, consisting of D-Box and E-Box sequences, the first controlled by light, and the second controlled by the clock. Consistent with the expression of *smper2* and *smcry1* being, at least in part, light regulated, peak levels of expression are observed soon after the onset of the light period. Furthermore, in all tissues tested, transfer to DD conditions resulted in an immediate, significant attenuation of rhythmic expression for these two genes with the only exception being *smper2* expression in the gut where the amplitude of rhythmic expression persisted and reduced more gradually. Interestingly, although *smper2* exhibited daily oscillations with a peak at ZT4 in the eye and hypothalamus, this peak was delayed 4-5h in muscle (ZT10) and intestine (ZT8), while in the liver *smper2* was arrhythmic. Similarly, for *smcry1* a phase delay of 2-3h was observed between central and peripheral tissues. Therefore, our data potentially points to the existence of a tissue specific interplay between light and clock regulation of these two clock genes. One key issue may be differences in the intensity of light penetrating deep tissues in the turbot body. An alternative possibility that would explain the difference in acrophases of *smper2* and *smcry1* between central and peripheral tissues might be the presence of an intermediate systemic signal that relays light information from the eye to central clocks and then in turn to the periphery. An important question is the degree of similarity between circadian clock function in turbot and the Senegalese sole, *Solea senegalensis* as well as other fish species. The expression profile of *smper2* from turbot differs partially from that described in the nocturnal *Solea*. In *Solea*, *per2* expression is arrhythmic in the retina, as well as in the liver. However, in the diencephalon it shows rhythmic expression with an acrophase at ZT5 [17]. In the turbot, *smper1* shows rhythmic expression in all tissues with an acrophase at the end of the night a profile that matches per1 expression in the *Solea* retina, but differs from that observed in the liver, where acrophase appears at the end of the day. The peak of *smclock1* expression in the retina and hypothalamus occurred at the end of the day (ZT9) and 2-3h later in muscle (ZT13), gut (ZT10) and liver (ZT11). In contrast, in the *Solea* retina, *Clock* expression peaks in the middle of the light phase (ZT6) and in the liver, in the middle of the dark phase (ZT18). However, the experimental conditions of the previous *Solea* study differ from those reported here for turbot. Thus, *Solea* were fed up to 3 times a day [17] compared with the single meal delivered daily in the current turbot study.

### Entrainment by food and light

Comparing rhythmic gene expression under LD with that observed with DD and regular feeding, the mesor and amplitude of all four clock genes were reduced by the lack of light. Therefore, although feeding time acts as a synchronizing factor, it is not sufficient to maintain the amplitude of the expression rhythms that seem to depend more strictly on the LD cycle. Interestingly, the phase of rhythmic clock gene expression under constant darkness observed in randomly fed and regularly fed animals was not significantly different with the exception of *smper1*and *smcry1*. A delay of 6h in the liver and 5h in the gut was observed in the expression of *smper1*. Furthermore, in the case of *smcry1* expression in the muscle we also observed a phase shift. These observations suggest that food entrains the expression of *smper1* in tissues closely related to the processing the food. However, under LD cycle conditions, the effect of light prevails over the food. Entrainment of the circadian expression of clock genes by food has also been demonstrated in mammals and other species of fish. The administration of food in the resting phase was shown to decouple the synchronization of clock gene expression rhythms in peripheral tissues (e.g. the liver) with central clocks (the SCN) [30].

In studies conducted in fish, the acrophases of clock genes in the liver of zebrafish, goldfish and tilapia were also affected depending of the feeding regimen [4,8,9].

While *Solea* activity is predominantly nocturnal, and locomotor activity rhythmicity is preserved in constant light or dark conditions [15,16], the swimming activity of turbot was mainly restricted to the light phase (Figs 4, 6 and 7), and under constant light or darkness it loses its rhythmicity. Our report is consistent with a previous study of, Champalbert and Le Direach-Bourssier [18] who had previously reported a loss of rhythmicity of turbot under constant darkness. The natural environment of turbot would tend to indicate that these fish might normally experience periods of complete darkness. Specifically, this flatfish’s habitats range from shallow waters down to a depth of 1500m [31] where very little light would penetrate through the water column. However, the absence of circadian rhythmicity under constant darkness may also relate to our experimental protocol. For example, typically bury themselves under sandy substrates so the absence of natural substrates in our experiments might contribute to the absence of rhythmicity. Furthermore, a masking effect of constant light or darkness upon endogenous activity levels cannot be ruled out. The light intensity used in our constant light experiments (380 lux) may also contribute to a masking effect. Indeed, in other species such as *Danio rerio*, rhythmic locomotor activity under free running conditions can only be visualized under constant dim lighting conditions.

In fish, the activity phase can be strongly influenced by the photoperiod, but also by the food administration phase although this observation does not seem to occur in all species-specific manner. Thus, while *Sparus aurata* synchronizes the activity phase with the feeding phase [32], in *Oreochromis niloticus*, which is predominantly diurnal, food failed to change the activity phase when it was delivered during the dark phase [8]. When fed regularly at the end of the night under a short photoperiod, similar to tilapia, turbot remains diurnal (Figs 7 and 8). Our results are consistent with the observations of Imsland et al. [19] which documented diurnal behaviour in juveniles under natural photoperiod and feeding during the photophase. Furthermore, under constant light the activity increased during the subjective night. In contrast, Champalbert and Direach-Boursier [18] identified a synchronizing effect of food on activity that depended on ontogeny. Thus, the larvae were arrhythmic in LD, but recently metamorphosed turbot were active during the scotophase. These authors also observed that juveniles fed in the morning becoming active during the photophase, whereas if they were older they became arrhythmic.

**Fig 9:**
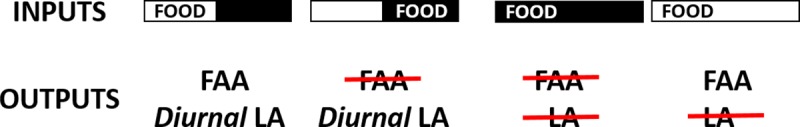
Summary outline from behavior experiments of the inputs regulation (scheduled feeding and light) of the outputs (Locomotor activity (LA) and FAA) in turbot juveniles under laboratory conditions. The rectangle represents the light phase (white) and the dark phase (black) within a period of 24h.

In our study we also observed a synchronizing effect of feeding time on the daytime swimming activity of juveniles fed each morning at the same time. A preprandial increase in locomotor activity (1.87-fold) was observed 2h before meal time (One-way ANOVA Kruskal-Wallis test, p <0.001), which subsequently declined to basal levels once the food in the water had been removed. Such behaviour was absent in those fish fed at random times. In DD conditions, turbot failed to anticipate the arrival of food, whereas in LL, the turbot juveniles exhibited FAA with a similar amplitude to that observed in LD. Furthermore, schedule feeding during the dark phase failed to entrain FAA. Our data indicate that turbot relies on light to establish rhythms of both locomotor activity and to enable FAA (Fig 9). Indeed, flatfish have been classified into three categories depending on their predatory strategy: visual feeders, predominantly visual feeders and non-visual nocturnal feeders. Holmes and Gibson [20,21] described *S*. *maximus* as an active visual predator to which moving and elongated objects incite a greater response than immobile and small objects such as artificial food pellets. This behaviour also correlates with the structure of the head and sensory organs [31]. This description indicates that light is a key signal to stimulate the search for food in turbot. In contrast, *Solea senegalensis* is a non-visual predator, and very sensitive to chemical substances such as betaine [20,33]. Indeed, *Solea* is predominantly nocturnal and food acts as a synchronizer of FAA rhythms in both the dark and light phases [15,16]. However, although turbot apparently cannot anticipate the arrival of food supplied regularly during darkness, it can still detect its presence in the water under these conditions.

The presence of FAA is interpreted as an output of a food entrainable oscillator or FEO [34]. At the molecular level, *per2* and *cry* genes have been linked with generating FAA. Thus, in mice expressing a *Per2* mutation specifically in the liver, FAA activity is abolished [35]. Furthermore, mutations of *per2* in zebrafish decrease activity in LD or even lead to arrhythmicity [36]. In our study, the hepatic expression of *smper2* was arrhythmic in all conditions studied, while in the hypothalamus *smper2* showed daily variations in LD but not in DD and daily fixed-time feeding (Fig 1 and Table 1).

Thus, differences in how light, feeding and the circadian clock differentially regulate clock gene expression as well as locomotor activity between the turbot and *Solea senegalensis* confirms the value of studying turbot as a comparative flatfish model. Furthermore, the knowledge gained should contribute to optimizing the aquaculture conditions for both species of flatfish.

## Supporting information

S1 Table Primer sequences used for cloning by RT-PCR and qPCR analysis of *per2*, cry1, *clock1* and *per1* expression in turbot. (DOCX)

S2 Table Species names, accession numbers from GenBank and length of amino acid sequences used to construct the phylogenetic tree (S1 Fig). (DOCX)

S1 Fig Phylogenetic tree of core clock proteins. The unrooted tree was generated in MEGA 5.1 using the Neighbor-joining method from the aligned amino acid sequences of PER2, PER1, CLOCK and CRY1 of mammals, amphibians, reptiles, birds and fish. The evolutionary distances were computed using the p-distance method. Branch lengths are in the same units as those of the evolutionary distances used to infer the phylogenetic tree. Bootstrap values were calculated from 10.000 permutations. The number next to each node indicates the percentage of times the subunits joined by the node were found in a monophyletic clade in the consensus tree of the bootstrap analysis. Scale bar indicates the amino acid changes per site. The analysis involved 39 amino acid sequences. Accession numbers are given in S2 Table.
(DOCX)

S2 Fig Alignment of full and partial (green) cDNA sequences of *clock*, *per1*, *per2* and *cry1* from *Scopthalmus maximus*. Identical nucleotides are indicated with asterisks, alignments were generated by using Clustal Omega (https://www.ebi.ac.uk/Tools/msa/clustalo/). Full sequences are available in Genbank and were deposited by Martinez, P with the following accession numbers: CP026254.1 for *smclock*, CP026244.1 for *smper1*, CP026245.1 for smper2 and CP026253.1 for smcry1. Partial sequences of clock genes were obtained in this study and accession numbers are in S2 Table.
(DOCX)
