# Supplementary material for: Differential circadian and light-driven rhythmicity of clock gene expression and behaviour in the turbot, Scophthalmus maximus
Source: PLoS One. 2019 Jul 5;14(7):e0219153. doi: 10.1371/journal.pone.0219153 (PMC6611576; doi:10.1371/journal.pone.0219153)
Supplement: S1 Table — (DOCX) [file pone.0219153.s001.docx]

**Supporting information**

S1 Table. Primer sequences used for cloning by RT-PCR and qPCR analysis of *per2*, *cry1*, *clock1* and *per1* expression in turbot.

| Target Gene | Primer | Sequence (5’ to 3’) | Amplicon (bp) | E (%) | Accession no. |
| --- | --- | --- | --- | --- | --- |
|  | **Real time RT-PCR** | |  |  |  |
| *Per2* | Fw | CTCGCTCATCACGGGAAAGAT | 98 | 93 | MH500050 |
|  | Rv | ACTCCACGAAATTGGCGTTGT |  |  |  |
| *ry1* | Fw | TGGACGCCAATTATGCACTTG | 67 | 108 | MH500051 |
|  | Rv | ACATCTACGACCCGTGGAACG |  |  |  |
| *Clock1* | Fw | CGTTGGATGGATTCTTCCTTGC | 132 | 105 | MH500052 |
|  | Rv | CCATTGGCAGGAAGTTCAACA |  |  |  |
| *Clock1 (for liver)* | Fw | CCTGTTGAACTTCCTGCCAATG | 82 | 109 | MH500052 |
|  | Rv | CAGCGTCTCTCCCTCCATGAT |  |  |  |
| *Per1* | Fw | GAGGGCCAGGATGAGGAGTTT | 110 | 90 | MH500049 |
|  | Rv | CACAGCTCCCTGGGAATGAAC |  |  |  |
| *Actin* | Fw | TGAACCCCAAAGCCAACAGG | 108 | 98 | AY008305.1 |
|  | Rv | CAGAGGCATACAGGGACAGCC |  |  |  |
| *Rpsd* | Fw | CGGAGGACGAGCAGGAA | 102 | 94 | [DQ848899](http://www.ncbi.nlm.nih.gov/entrez/query.fcgi?cmd=search&db=nucleotide&doptcmdl=genbank&term=DQ848899) |
|  | Rv | TGCGGACGGCAGTGATG |  |  |  |
| *Rpl8* | Fw | CTCCGCCACATTGACTTC | 197 | 101.2 | [DQ848874](http://www.ncbi.nlm.nih.gov/entrez/query.fcgi?cmd=search&db=nucleotide&doptcmdl=genbank&term=DQ848874) |
|  | Rv | GCCTTCTTGCCACAGTAG |  |  |  |
|  | **Cloning RT-PCR** | |  |  |  |
| *Per2* | Fw | CATCACGTCTGAGTACACCCT | 870 |  |  |
|  | Rv | AGCAGCCTGTGGATCTGTTCACT |  |  |  |
| *Clock1* | Fw | CGTTGGATGGATTCTTCCTTGC | 851 |  |  |
|  | Rv | TGCCATCTCAGGTGGTGATTC |  |  |  |
| *Cry1* | Fw | GGCAGCCTCCTCTCACCTACA | 964 |  |  |
|  | Rv | TGGACGCCAATTATGCACTTG |  |  |  |
| *Per1* | Fw | CGTTCTCCTTGGACGTATCATA | 798 |  |  |
|  | Rv | GAGTTCGCTGTTCCCGTGACT |  |  |  |
