# Supplementary material for: Differential circadian and light-driven rhythmicity of clock gene expression and behaviour in the turbot, Scophthalmus maximus
Source: PLoS One. 2019 Jul 5;14(7):e0219153. doi: 10.1371/journal.pone.0219153 (PMC6611576; doi:10.1371/journal.pone.0219153)
Supplement: S2 Table — (DOCX) [file pone.0219153.s002.docx]

**Supporting information**

S2 Table. Species names, accession numbers from GenBank and length of amino acid sequences used to construct the phylogenetic tree (S1 Fig).

| **PROTEIN** | **SPECIES** | **GENBANK ACCESSION NO.** | **LENGTH (aa)** |
| --- | --- | --- | --- |
| **PER1** | *Homo sapiens* | AAF15544.1 | 1290 |
|  | *Mus musculus* | BAA94086.1 | 1291 |
|  | *Anolis carolinensis* | XP_008117781.2 | 1315 |
|  | *Xenopus laevis* | NP_001079172.2 | 1234 |
|  | *Solea senegalensis* | CAQ81976.1 | 238 (partial) |
|  | *Danio rerio* | NP_997604.2 | 1398 |
|  | *Paralichthys olivaceus* | XP_019951105.1 | 1437 |
|  | *Takifugu rubripes* | XP_011604999.1 | 1421 |
|  | *Dicentrarchus labrax* | ADI71975.1 | 1436 |
|  | *Scophthalmus maximus* | MH500049 | 265 (partial) |
| **PER2** | *Homo sapiens* | NP_073728.1 | 1255 |
|  | *Mus musculus* | NP_035196.2 | 1257 |
|  | *Gallus gallus* | NP_989593.1 | 1344 |
|  | *Anolis carolinensis* | XP_008104509.1 | 1284 |
|  | *Xenopus laevis* | NP_001081098.1 | 1427 |
|  | *Solea senegalensis* | CAQ86911.1 | 609 (partial) |
|  | *Danio rerio* | NP_878277.2 | 1399 |
|  | *Paralichthys olivaceus* | XP_019950584.1 | 1503 |
|  | *Takifugu rubripes* | XP_011607058.1 | 1502 |
|  | *Scophthalmus maximus* | MH500050 | 289 (partial) |
| **CRY1** | *Homo sapiens* | NP_004066.1 | 586 |
|  | *Mus musculus* | NP_031797.1 | 606 |
|  | *Gallus gallus* | AAK61385.1 | 621 |
|  | *Anolis carolinensis* | XP_003220970.1 | 621 |
|  | *Xenopus laevis* | NP_001081129.1 | 616 |
|  | *Danio rerio* | NP_001300751.1 | 658 |
|  | *Paralichthys olivaceus* | XP_019966660.1 | 592 (partial) |
|  | *Takifugu rubripes* | XP_011607950.1 | 527 |
|  | *Scophthalmus maximus* | MH500051 | 320 (partial) |
| **CLOCK1** | *Homo sapiens* | XP_016864343.1 | 846 |
|  | *Mus musculus* | NP_001276755.1 | 855 |
|  | *Gallus gallus* | NP_001276763.1 | 852 |
|  | *Anolis carolinensis* | XP_008109890.1 | 887 |
|  | *Xenopus laevis* | NP_001083854.2 | 825 |
|  | *Danio rerio* | NP_840080.2 | 820 |
|  | *Paralichthys olivaceus* | XP_019937002.1 | 922 |
|  | *Takifugu rubripes* | XP_011612850.1 | 860 |
|  | *Scophthalmus maximus* | MH500052 | 283 (partial) |
