# Supplementary material for: Differential circadian and light-driven rhythmicity of clock gene expression and behaviour in the turbot, Scophthalmus maximus
Source: PLoS One. 2019 Jul 5;14(7):e0219153. doi: 10.1371/journal.pone.0219153 (PMC6611576; doi:10.1371/journal.pone.0219153)
Supplement: S1 Fig — The unrooted tree was generated in MEGA 5.1 using the Neighbor-joining method from the aligned amino acid sequences of PER2, PER1, CLOCK and CRY1 of mammals, amphibians, reptiles, birds and fish. The evolutionary distances were computed using the p-distance method. Branch lengths are in the same units as those of the evolutionary distances used to infer the phylogenetic tree. Bootstrap values were calculated from 10.000 permutations. The number next to each node indicates the percentage of times the subunits joined by the node were found in a monophyletic clade in the consensus tree of the bootstrap analysis. Scale bar indicates the amino acid changes per site. The analysis involved 39 amino acid sequences. Accession numbers are given in S2 Table. (DOCX) [file pone.0219153.s003.docx]

**Supporting information**


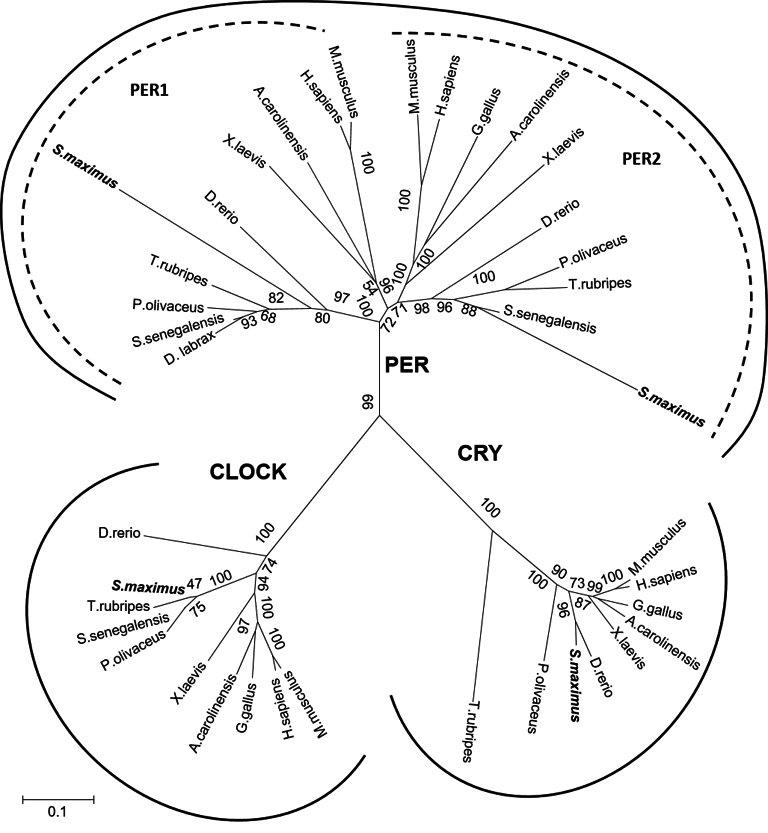


S1 Fig. Phylogenetic tree of core clock proteins. The unrooted tree was generated in MEGA 5.1 using the Neighbor-joining method from the aligned amino acid sequences of PER2, PER1, CLOCK and CRY1 of mammals, amphibians, reptiles, birds and fish. The evolutionary distances were computed using the p-distance method. Branch lengths are in the same units as those of the evolutionary distances used to infer the phylogenetic tree. Bootstrap values were calculated from 10.000 permutations. The number next to each node indicates the percentage of times the subunits joined by the node were found in a monophyletic clade in the consensus tree of the bootstrap analysis. Scale bar indicates the amino acid changes per site. The analysis involved 39 amino acid sequences. Accession numbers are given in S2 Table.
