# Supplementary material for: Differential circadian and light-driven rhythmicity of clock gene expression and behaviour in the turbot, Scophthalmus maximus
Source: PLoS One. 2019 Jul 5;14(7):e0219153. doi: 10.1371/journal.pone.0219153 (PMC6611576; doi:10.1371/journal.pone.0219153)
Supplement: S2 Fig — Identical nucleotides are indicated with asterisks, alignments were generated by using Clustal Omega (https://www.ebi.ac.uk/Tools/msa/clustalo/). Full sequences are available in Genbank and were deposited by Martinez, P with the following accession numbers: CP026254.1 for smclock, CP026244.1 for smper1, CP026245.1 for smper2 and CP026253.1 for smcry1. Partial sequences of clock genes were obtained in this study and accession numbers are in S2 Table. (DOCX) [file pone.0219153.s004.docx]

**Supporting information**

S2 Fig. Alignment of full and partial (green) cDNA sequences of *clock*, *per1*, *per2* and *cry1* from *Scophthalmus* *maximus*. Identical nucleotides are indicated with asterisks, alignments were generated by using Clustal Omega (<https://www.ebi.ac.uk/Tools/msa/clustalo/>). Full sequences are available in Genbank and were deposited by Martinez, P with the following accession numbers: CP026254.1 for *smclock*, CP026244.1 for *smper1*, CP026245.1 for *smper2* and CP026253.1 for *smcry1*. Partial sequences of clock genes were obtained in this study and accession numbers are presented in S2 Table.

**Clock**

CP026254.1 ATGAAGCTTTTACCTGCAAACTGTATAATGAAAAATGTGTATTGCATCTGTATCCTCAGC 60

Partial_clock ------------------------------------------------------------ 0

CP026254.1 GTGTCACGTAACAAGTCTGAGAAGAAACGCAGAGACCAGTTCAATGTCCTCATCAAGGAG 120

Partial_clock ------------------------------------------------------------ 0

CP026254.1 CTGGGTACAATGTTACCGGGCAACACCCGGAAAATGGACAAGTCCACTATTTTGCAGCAG 180

Partial_clock ------------------------------------------------------------ 0

CP026254.1 AGCATCGACTATCTGCACAAACACAAGGAAATCACTGCTCAGTCGGAGTCAACTGAGATC 240

Partial_clock ------------------------------------------------------------ 0

CP026254.1 AGACAAGACTGGAAGCCTCCTTTTCTTAGTAATGAAGAGTTCACTCAGCTGATGTTGGAG 300

Partial_clock ------------------------------------------------------------ 0

CP026254.1 GCGTTGGATGGATTCTTCCTTGCAATTATGACGGATGGGAATATAATCTATGTCTCTGAG 360

Partial_clock -CGTTGGATGGATTCTTCCTTGCAATTATGACGGATGGGAATATAATCTATGTCTCTGAG 59

***********************************************************

CP026254.1 AGCGTGACGTCTCTACTAGAACATTTTCCTTCTGATCTTGTTGATCAGAACCTGTTGAAC 420

Partial_clock AGCGTGACGTCTCTACTAGAACATTTTCCTTCTGATCTTGTTGATCAGAACCTGTTGAAC 119

************************************************************

CP026254.1 TTCCTGCCAATGGGGGAGCATTCAGACGTGTACAAGGCTCTGTCCTCTCATATCATGGAG 480

Partial_clock TTCCTGCCAATGGGGGAGCATTCAGACGTGTACAAGGCTCTGTCCTCTCATATCATGGAG 179

************************************************************

CP026254.1 GGAGAGACGCTGACACCTGAGTATCTGAAAACAAAAAATCAGCTAGAGTTCTGTTGCCAC 540

Partial_clock GGAGAGACGCTGACACCTGAGTATCTGAAAACAAAAAATCAGCTAGAGTTCTGTTGCCAC 239

************************************************************

CP026254.1 ATGCTCCGAGGGACCATCCACCCTAAGGAGCCCCCCGTGTACGAGTATGTCAAGTTCATT 600

Partial_clock ATGCTCCGAGGGACCATCCACCCTAAGGAGCCCCCCGTGTACGAGTATGTCAAGTTCATT 299

************************************************************

CP026254.1 GGCAACTTCAAGTCCCTGAATAATGTGCCTAACTGTACCCAAAACGGTTTTGAAGGAGTG 660

Partial_clock GGCAACTTCAAGTCCCTGAATAATGTGCCTAACTGTACCCAAAACGGTTTTGAAGGAGTG 359

************************************************************

CP026254.1 ATCCAGCGATCACTTGTTCACTCTGCCTTCGAAGACAGAGTGTGTCTCATAGCAACTGTG 720

Partial_clock ATCCAGCGATCACTTGTTCACTCTGCCTTCGAAGACAGAGTGTGTCTCATAGCAACTGTG 419

************************************************************

CP026254.1 AGGCTCGCCAAACCACAGTTCATCAAGGAGATGTGCACTGTTGAAGAGCCTAATGAGGAA 780

Partial_clock AGGCTCGCCAAACCACAGTTCATCAAGGAGATGTGCACTGTTGAAGAGCCTAATGAGGAA 479

************************************************************

CP026254.1 TTCACCTCCAGACATAGTTTAGAGTGGAAATTTCTCTTCTTGGACCACAGAGCTCCACCC 840

Partial_clock TTCACCTCCAGACATAGTTTAGAGTGGAAATTTCTCTTCTTGGACCACAGAGCTCCACCC 539

************************************************************

CP026254.1 ATCATAGGTTACCTCCCGTTTGAGGTCCTGGGTACATCAGGATATGACTACTACCATGTA 900

Partial_clock ATCATAGGTTACCTCCCGTTTGAGGTCCTGGGTACATCAGGATATGACTACTACCATGTA 599

************************************************************

CP026254.1 GATGACCTGGAGACACTAGCCAAATGCCATGAACACTTAATGCAATACGGTAAAGGAAAG 960

Partial_clock GATGACCTGGAGACACTAGCCAAATGCCATGAACACTTAATGCAATACGGTAAAGGAAAG 659

************************************************************

CP026254.1 TCCTGCTACTACAGATTCCTCACAAAAGGGCAGCAGTGGATTTGGCTTCAAACCCACTAC 1020

Partial_clock TCCTGCTACTACAGATTCCTCACAAAAGGGCAGCAGTGGATTTGGCTTCAAACCCACTAC 719

************************************************************

CP026254.1 TACATCACCTACCACCAGTGGAACTCCAGACCAGAGTTTATTGTCTGCACACACACTGTT 1080

Partial_clock TACATCACCTACCACCAGTGGAACTCCAGACCAGAGTTTATTGTCTGCACACACACTGTT 779

************************************************************

CP026254.1 GTCAGTTACGCTGAAGTAAGAGCAGAACAGCGCAGAGAACTTGGAATTGAAGAATCACCA 1140

Partial_clock GTCAGTTACGCTGAAGTAAGAGCAGAACAGCGCAGAGAACTTGGAATTGAAGAATCACCA 839

************************************************************

CP026254.1 CCTGAGATGGCAGTAGATAAGTCCCAGAATTCGGGCTCCGAGTCCCAACTCAACACTTCC 1200

Partial_clock CCTGAGATGGCA------------------------------------------------ 851

************

CP026254.1 AGTCTGAAGGAGGTCTTGGAGCGATTTGACCACAGCCGGACGCCCTCGGTTTTGTCTCGC 1260

Partial_clock ------------------------------------------------------------ 851

CP026254.1 AGCTCCCACAAGTCATCACACACTGCTGCGTCTGACCCAGCCTCATCACAGATGAAGCTT 1320

Partial_clock ------------------------------------------------------------ 851

CP026254.1 CAGGGAGATAGGAGCACACCGGGTCGCCAGTCTGTCTCTGCTGTGGAGATGACGTCACAA 1380

Partial_clock ------------------------------------------------------------ 851

CP026254.1 CGAAGATCATCTATCAGCAGTCAGCACTCGATGAGCTCCCAAAATACCGGACAAAACATC 1440

Partial_clock ------------------------------------------------------------ 851

CP026254.1 ACTCCATCCACGGTCTCTCAACAACAACCACAACAGCAACAGCCTCAACAGCAACAAGTT 1500

Partial_clock ------------------------------------------------------------ 851

CP026254.1 CAAAACAATGGACAATCAATGGTGCAGTTCTCAGGCCAGTTGGAAGCCATGCATCACCTG 1560

Partial_clock ------------------------------------------------------------ 851

CP026254.1 AAAGAGAAGCTGGAGCAGAGGACCAGGATGATCGAGGCCAACATCCAGCTGCAGCAGGAC 1620

Partial_clock ------------------------------------------------------------ 851

CP026254.1 GAGCTGCGACAGATTCAGGATGAGCTGAATAAGGTGCAGGGACAGAACCTGCAGATGATC 1680

Partial_clock ------------------------------------------------------------ 851

CP026254.1 TTGCAGAAAGGAGCTGGAGGACTTACTGTTGGCTCTGTCCAGATGGCCCAGGGGAACGTT 1740

Partial_clock ------------------------------------------------------------ 851

CP026254.1 GTGCAGCAGGGGGCGACGCTCAGCATGCAGGGTCAGGTTGTCTCTGCGGGGCCTTTACAG 1800

Partial_clock ------------------------------------------------------------ 851

CP026254.1 AACAGTATAAAGCAGCAACATGCTGTCCAGCCCCAATCCCAGCAACAGACACTCTCTCAG 1860

Partial_clock ------------------------------------------------------------ 851

CP026254.1 TGTCAACGGTCGTCCCTCACTCTGCAGCCTCAGCAAAACACACTTCCCATGTCTCTCTAC 1920

Partial_clock ------------------------------------------------------------ 851

CP026254.1 AACACGATGATGATCCCTCAGCAAAGCCCCGCTAACGTGATGCAGATTGCCACTAGCCTG 1980

Partial_clock ------------------------------------------------------------ 851

CP026254.1 GCACAGAACACTGGACACAACACTCAAGCGGTGGCAACATTTGCACAGGACCGTGCTGCT 2040

Partial_clock ------------------------------------------------------------ 851

CP026254.1 CAGATGAGGCTCCTCGGCTTCTTCATGGAAACCAGTCCACCCAGTTGA 2088

Partial_clock ------------------------------------------------ 851

**Per1**

CP026244.1 ATGAGTTATGACAACTCTAAATCAATGCCCAGCAGCAGCAGCACTCGGGGGCGAGTGTCA 60

Partial_per1 ------------------------------------------------------------ 0

CP026244.1 AGGGCCGACGGGAATGACAATGACCAGGAGGCAGAGTCAGAAGGGCTGAACTCACCAGAA 120

Partial_per1 ------------------------------------------------------------ 0

CP026244.1 ACCATCGGTAGTCATTCCAGTGCCAATGTCGCCACCCTGGAGCAGGGACGCGGAGCCGGG 180

Partial_per1 ------------------------------------------------------------ 0

CP026244.1 GGAGACTCGTCTCCCAGCGGAGGGTCGGGTTCTGGAGGCTCATCCAGAGACCAAAGGGGG 240

Partial_per1 ------------------------------------------------------------ 0

CP026244.1 CCCAACTCTGATGATATGGACGGCCTCTCCAGCGGGAACGACTCCGGGGAGCGGGAAAGC 300

Partial_per1 ------------------------------------------------------------ 0

CP026244.1 GAGAGGGGGAACGGGTCACGCGGGCGCCAGTCCATACGCAGCTCCCACAGCTCTTCGAAC 360

Partial_per1 ------------------------------------------------------------ 0

CP026244.1 GGCAAGGACTCTGGCATGATGCTGGAAACCACAGAGAGCAACAAGAGCTCCAACTCCCAG 420

Partial_per1 ------------------------------------------------------------ 0

CP026244.1 AGCCTCTCACCTCCCAGCAGCTCCGTGGCCTACAGCCTGATGTCAACCAACTTGGAGCAC 480

Partial_per1 ------------------------------------------------------------ 0

CP026244.1 GACCCTCCCTCCACCTCCGGGTGTAGCAGCAACCAGTCAGCGAGGATCCAGACCCAGAAA 540

Partial_per1 ------------------------------------------------------------ 0

CP026244.1 GAGCTCATGAAGGCTATCAAGGAGCTGAAGCTCCGCCTGCCGTCTGAGCGCAAGGCCAAG 600

Partial_per1 ------------------------------------------------------------ 0

CP026244.1 GGACACTCCAGCACACTCAATGCACTCAAATACGCACTTCAGTGTGTCAGACAAGTCAGA 660

Partial_per1 ------------------------------------------------------------ 0

CP026244.1 GCTAACCGGGAGTACTACCACCAGTGGAGTGTGGAGGAGTGTCACGGCTGCAGTCTGGAC 720

Partial_per1 ------------------------------------------------------------ 0

CP026244.1 CTGTCAGCCTTCACAATCGAGGAGCTTGATAACATCACCTCGGAGTACACCCTCAAAAAC 780

Partial_per1 ------------------------------------------------------------ 0

CP026244.1 ACTGACACGTTCACCATGGCCGTGTCTTTCTTGACAGGGAAGGTCGTGTACGTATCACCC 840

Partial_per1 ------------------------------------------------------------ 0

CP026244.1 CAGGGCTCGTCCCTGCTGCGCTGTAAGCCCGAGTTTCTCCAGGGGATTATGTTTTCGGAG 900

Partial_per1 ------------------------------------------------------------ 0

CP026244.1 CTTTTGGCCCCGCAGGATGTCAGTACTTTCTACAGCGGCACAGCACCCTGCCGCCTTCCT 960

Partial_per1 ------------------------------------------------------------ 0

CP026244.1 TCCTGGGCCTCCTGCATCGGATCTGCACCTCCTCCAGTCGATTGCACTCAAGAGAAGTCC 1020

Partial_per1 ------------------------------------------------------------ 0

CP026244.1 ATGTTCTGTCGGATCCGAGCTGACCGGGCACACGGTGGCGAGATGCGCTACTACCCATTC 1080

Partial_per1 ------------------------------------------------------------ 0

CP026244.1 CGCCTCACGCCATACCAGCTCACTCTTAGGGACTTTGATGCCGCAGAGCCACAGCCCTGC 1140

Partial_per1 ------------------------------------------------------------ 0

CP026244.1 TGCCTGCTCATCGCAGAGAGGGTCCACTCTGGATATGAGGCTCCTCGTATCCCAGCAGAC 1200

Partial_per1 ------------------------------------------------------------ 0

CP026244.1 AAGAGGATCTTCACCACCAGTCACACTCCCAGCTGCCTCTTCCAGGAGGTGGATGAGAGG 1260

Partial_per1 ------------------------------------------------------------ 0

CP026244.1 GCGGTGCCATTGTTGGGCTACCTGCCTCAGGACTTGGTGGGAACCCCCACCCTGCTCTAC 1320

Partial_per1 ------------------------------------------------------------ 0

CP026244.1 ATCCACCCCGAGGACAGGCCCATGATGGTGGCCATACATGAGAAGATCTTTCAGTCTGCC 1380

Partial_per1 ------------------------------------------------------------ 0

CP026244.1 GGGCAGCCGTTTGACTATTCCCCCATGAGGATGTGTGCCCGCAGCGGGGAGTATCTGACC 1440

Partial_per1 ------------------------------------------------------------ 0

CP026244.1 ATTGACACCAGCTGGTCGTCCTTTGTCAACCCCTGGAGCCGCAAGGTGGCCTTCATCGTA 1500

Partial_per1 ------------------------------------------------------------ 0

CP026244.1 GGGCGCCACAAAGTCAGAACGAGCCCTCTGAATGAAGACGTGTTCACTATGCCGCAAGGC 1560

Partial_per1 ------------------------------------------------------------ 0

CP026244.1 TGCGAGGACCGCGTCACCACGCCCGACATCGTCCAGCTGAGCGAGCGGATCCACCGGGTC 1620

Partial_per1 ------------------------------------------------------------ 0

CP026244.1 CTGGTGCAGCCAGTGCACAGCGGCAGCTCCCAGGGATACTGCTCCCTTGGGTCCAGTGGG 1680

Partial_per1 ------------------------------------------------------------ 0

CP026244.1 TCGCGGGGCTCCCGCCGGTCTCACCAACAGCACCTTAGTGCATCGGCCGGCTCGTCCAGC 1740

Partial_per1 ------------------------------------------------------------ 0

CP026244.1 GACAGCAACGGCCCCGCCATGGACGTGGCTGCCACTGCCGCCGTCGCTTTACACAAACCC 1800

Partial_per1 ------------------------------------------------------------ 0

CP026244.1 ATGACGTTCCAGCAGATCTGCAAAGACGTTCACATGGTCAAGACGAACGGGCAGCAGGTT 1860

Partial_per1 ------------------------------------------------------------ 0

CP026244.1 TTCATCGAGTCCCGCAACCGTCCGGTGCCCAGAAAAAACATCAGCGCAGGCACAACAAGC 1920

Partial_per1 ------------------------------------------------------------ 0

CP026244.1 ATCAGAGCGATCAGCAGTGACCCAATCAGAGGTTTGATAGCAGACTTGACCAAACCGCTT 1980

Partial_per1 ------------------------------------------------------------ 0

CP026244.1 AAAGCTTTGGTCCCTGCTCCTCTTGTACAGAAGGAGCCGCCGCCCGGCTACTCCTACCAG 2040

Partial_per1 ------------------------------------------------------------ 0

CP026244.1 CAGATCAACTGTCTGGACAGCATCATACGTTACTTGGAGGGTTGCAACATTCCCAACACG 2100

Partial_per1 ------------------------------------------------------------ 0

CP026244.1 GTGAAAAGGAAGTGTGGCTCCTACACGACCTCCTCCACGTCTGATGACGACAAACAGCAG 2160

Partial_per1 ------------------------------------------------------------ 0

CP026244.1 GAGACCATCAATAATAGCAGAGGTGGTTCAGTTAGCCTCGTAGGTGAACCACCTACTCTG 2220

Partial_per1 ------------------------------------------------------------ 0

CP026244.1 CCCCCCTTGACCATGGCCACAAAGGCAGAGAGTGTAGCCTCAGTCACGTCCCAGTGTAGC 2280

Partial_per1 ------------------------------------------------------------ 0

CP026244.1 TTCAGCAGCACCATCGTGCATGTGGGAGACAAGAAGCCTCCCGAGTCAGACATCGTCATG 2340

Partial_per1 ------------------------------------------------------------ 0

CP026244.1 GAAGACGCTCCTACAACTCACACTCTGGCCGCTCCTCTGACTACTGCTGCCACAGCGCCG 2400

Partial_per1 ------------------------------------------------------------ 0

CP026244.1 CCGCCTCCTCCTCCCCCACCAACCCCTCCCCTGCCTCCCTTCCCACAAGCCACTCAGCCG 2460

Partial_per1 ------------------------------------------------------------ 0

CP026244.1 GAAAGGGACAGCAGGAGGTGCGGAAGTGTCGGAGGAGGTCGGCTGGGTCTGACCAAGGAG 2520

Partial_per1 ------------------------------------------------------------ 0

CP026244.1 GTGCTCTCCGCCCACACCCAGCAGGAGGAGCAGGCGTTCCTTGACCACTTCAAGGACCTC 2580

Partial_per1 ------------------------------------------------------------ 0

CP026244.1 AGCAAGCTGCGTGTGTTCGATCAGACGGCGTCTTCGACCGTGCGATGCAACACCCCAACT 2640

Partial_per1 ------------------------------------------------------------ 0

CP026244.1 GCCAACCCTCTGTCACGAGGAGTTCGCTGTTCCCGTGACTACCCCGCTGCAGGAAGCAGC 2700

Partial_per1 -------------------GAGTTCGCTGTTCCCGTGACTACCCCGCTGCAGGAAGCAGC 41

*****************************************

CP026244.1 AATGGTCACAGGCGGGGCCGTGGAGGAAAGAGACTCAAGCACCAGGAGTCTTCAGACCAA 2760

Partial_per1 AATGGTCACAGGCGGGGCCGTGGAGGAAAGAGACTCAAGCACCAGGAGTCTTCAGACCAA 101

************************************************************

CP026244.1 CACAGCTCCCTGGGAATGAACGGGAGCCGCCAAGACCTCAGGACCAGCACCGCCCCAATG 2820

Partial_per1 CACAGCTCCCTGGGAATGAACGGGAGCCGCCATGACCTCAGGACCAGCACCGCCCCAATG 161

******************************** ***************************

CP026244.1 CCCCTCAACATGCCTCTGGCACCCCCGACAAACTCCTCATCCTGGCCCTCCGTTCACTCT 2880

Partial_per1 CCCCTCAACATGCCTCTGGCACCCCCGACAAACTCCTCATCCTGGCCCTCCGTTCACTCT 221

************************************************************

CP026244.1 CAGGCCAGCATGCCCGCCGCCCCTTTCGCTCCCGGTATGCTTCCACTCTACCCCGTTTAC 2940

Partial_per1 CAGGCCAGCATGCCCGCCGCCCCTTTCGCTCCCGGTATGCTTCCACTCTACCCCGTTTAC 281

************************************************************

CP026244.1 CCACCGCTCGCGCAGCCCTTACCAATCCCCCCTACCCAGATGGTTCCTCCCATGATGGCC 3000

Partial_per1 CCACCGCTCGCGCAGCCCTTACCAATCCCCCCTACCCAGATGGTTCCTCCCATGATGGCC 341

************************************************************

CP026244.1 CTCGTTCTGCCCCCATACATGTTCCCCCAGATGGGAGCACCCCTCCCTCAGCCGGGTGCC 3060

Partial_per1 CTCGTTCTGCCCCCATACATGTTCCCCCAGATGGGAGCACCCCTCCCTCAGCCGGGTGCC 401

************************************************************

CP026244.1 ACCCCTGGACACTTCTACAACCCGAACTTCGCGTACCCCGGTGCTACCCCAGCTGTTCAC 3120

Partial_per1 ACCCCTGGACACTTCTACAACCCGAACTTCGCGTACCCCGGTGCTACCCCAGCTGTTCAC 461

************************************************************

CP026244.1 GCAGCCATTCACCCGGCCGCCATCCCGGCCGCCGTCCCCTCTATTGTCTCTCACCCGATG 3180

Partial_per1 GCAGCCATTCACCCGGCCGCCATCCCGGCCGCCGTCCCCTCTATTGTCTCTCACCCGATG 521

************************************************************

CP026244.1 CCCATTCCCGGCAACTGCGCCCCGTCTCGTAGCAGCACCCCGCACTCTTACAGCCAGATG 3240

Partial_per1 CCCATTCCCGGCAACTGCGCCCCGTCTCGTAGCAGCACCCCGCACTCTTACGGCCAGATG 581

*************************************************** ********

CP026244.1 CCCGCTGACCGGGAGGGGGCAGAGTCCCCCCTCTTCCAGTCCCGATGCTCCTCCCCACTC 3300

Partial_per1 CCCGCTGACCGGGAGGGGGCAGAGTCCCCCCTCTTCCAGTCCCGATGCTCCTCCCCACTC 641

************************************************************

CP026244.1 AACTTGTTGCAGCTGGAGGAGACGCCATGTAACCGTCTAGAGGTTGCCACGGCGCTGGCG 3360

Partial_per1 AACTTGTTGCAGCTGGAGGAGACGCCATGTAACCGTCTAGAGGTTGCCCCGGCGCTGGCG 701

************************************************ ***********

CP026244.1 GCATCACAGCAGGCCGCGCCTTCTGTGCAGGGCAGTGCAGCCGGGGGCCCGAGCTCCGCC 3420

Partial_per1 GCATCACAGCAGGCCGCGCCTTCTGTGCAGGGCAGTGCAGCCGGGGGCCCGAGCTCCGCC 761

************************************************************

CP026244.1 ACTCAGAGGAGCTCTGATGATACGTCCAAGGAGAACGAGAATGGTGAAACGAACGAGTCA 3480

Partial_per1 ACTCAGAGGAGCTCTGATGATACGTCCAAGGAGAACG----------------------- 798

*************************************

CP026244.1 AACAACGACGCCATGTCCACCTCCAGCGACCTGCTGGACCTGCTGCTGCAGGAGGACTCC 3540

Partial_per1 ------------------------------------------------------------ 798

CP026244.1 CGCTCAGGCACCGGCTCAGCTGGCTCTGGTTCAGGTTTCTCGGGAACGAGGTCCTCGGGT 3600

Partial_per1 ------------------------------------------------------------ 798

CP026244.1 TCCGGCTCCGGCTCAAACGGTTGCACCAGTGGCACCAGCAGCAGTCAGGGCAGCCACACC 3660

Partial_per1 ------------------------------------------------------------ 798

CP026244.1 AGCAAGTACTTTGGCAGCATCGACTCATCAGAGAACGACCACTCCCGCAAACAGCCAGCA 3720

Partial_per1 ------------------------------------------------------------ 798

CP026244.1 GGGGGCAGCAGCAGCACTGGGGGCGAGGAGCAGTTCATCAAGTGTGTCCTCCAGGACCCA 3780

Partial_per1 ------------------------------------------------------------ 798

CP026244.1 ATCTGGCTACTCATGGCCAACACCGACGACAAGGTCATGATGACCTATCAGCTGCCTGTC 3840

Partial_per1 ------------------------------------------------------------ 798

CP026244.1 AGGGACATGGAGACGGTGCTGCGCGAGGACCGCGAGGCCTTGAGGAGCATGCAGAAACAC 3900

Partial_per1 ------------------------------------------------------------ 798

CP026244.1 CAGCCACGCTTCACCGAGGAGCAGAAGAGGGAGCTGAGCCAGGTTCACCCCTGGATCCGC 3960

Partial_per1 ------------------------------------------------------------ 798

CP026244.1 ACAGGACGCCTGCCGCGAGCCATCAACATCTCTGGCTGCACGGACTGCAAGTCCCCCCCC 4020

Partial_per1 ------------------------------------------------------------ 798

CP026244.1 ACTGTGCCACCGGCCGCCCCGTTCGACGTGGAGATCCACGAGATGGAGCTGTGCAGTGTG 4080

Partial_per1 ------------------------------------------------------------ 798

CP026244.1 CTGAAGGCTCAGGAGGAGGGCGCCAAGAAGAATCTCTCCGAAACGGCCATGGACGAAACT 4140

Partial_per1 ------------------------------------------------------------ 798

CP026244.1 CACGCAGAGGATGAAGACGACGAGGGGGAGGAGAAAGACACCAAAACGCAAGACAGCAAC 4200

Partial_per1 ------------------------------------------------------------ 798

CP026244.1 CACGACATGACGGCAGAGGAGCCGAGAGTGTCCTCAGCCGCTGTGGAGGCGAAGGCTCAG 4260

Partial_per1 ------------------------------------------------------------ 798

CP026244.1 TCGGCCGAGTCTGACATGACTCAGTAG 4287

Partial_per1 --------------------------- 798

**Per2**

CP026245.1 ATGTCTGAGGACAGCGATCCCAATCACTATCTGTACTCAACCCTGGACGGTCCTGAGCGG 60

Partial_per2 ------------------------------------------------------------ 0

CP026245.1 AACCGGGGAAGGGTCGACTTGAACGCGGAGGAAAGGGAGGGCTCACCCTGCGGCGCCATT 120

Partial_per2 ------------------------------------------------------------ 0

CP026245.1 AGCCAACTTCGCCACATGGCCAGCGGTTACAGCGAGGGTTGTGGCGGGCCGGATGGGGTC 180

Partial_per2 ------------------------------------------------------------ 0

CP026245.1 GAACTGGAGCCAGAGTTGGGCCTGGCCTCCGAGGGGAGCGAAAGCAGTCACGAGCGCCCG 240

Partial_per2 ------------------------------------------------------------ 0

CP026245.1 ACCTCGCCGCACGACGACCGGAAGCGGCAGCGCTCGGCCTTGCACGAAGACATGGAGATG 300

Partial_per2 ------------------------------------------------------------ 0

CP026245.1 GGCGGCAGCGGTTCAAGCGGCAGCGGGACAGAGTCCCACGGGAATGAGTCGCACGGCAAC 360

Partial_per2 ------------------------------------------------------------ 0

CP026245.1 GAGTCCCACGGCAACGAATCTGTCGGCAGCTCGAGTGGCAACGGCAAGGATTCGGCTCTT 420

Partial_per2 ------------------------------------------------------------ 0

CP026245.1 ATGGAGTCTCTGGGGAGCACCAAGAGCTCAAACTCGCACAGCCCGTCGCCCCCGAGCAGC 480

Partial_per2 ------------------------------------------------------------ 0

CP026245.1 TCCAACGCCTTCAGTCTGGTGAGCTCGGAGCAGGACAACCCGTCGACGAGCGGCTGCAGC 540

Partial_per2 ------------------------------------------------------------ 0

CP026245.1 AGCGAGCAGTCGGCCAAAGCGAAGACTCAGAAGGAGCTGTTCAAGACCCTCAAGGAGCTG 600

Partial_per2 ------------------------------------------------------------ 0

CP026245.1 AAGACGCACCTGCCGTCGGAGAAAAGGAGCAAGGGCAAGTCCAGCACCATCAACACGCTC 660

Partial_per2 ------------------------------------------------------------ 0

CP026245.1 AAGTATGCGCTGCGATGTGTCAAACAGGTGAAAGCCAATGAGGAATACTACCAGATGCTG 720

Partial_per2 ------------------------------------------------------------ 0

CP026245.1 ATGGTTAATGACAGTCAGCCTCCGGGGTTCGATGTGTCGTCCTACACCCTCGAGGAAATC 780

Partial_per2 ------------------------------------------------------------ 0

CP026245.1 AACCGCATCACGTCTGAGTACACCCTGAAAAACACAGATATATTTGCCGTAGCCGTCTCG 840

Partial_per2 -----CATCACGTCTGAGTACACCCTGAAAAACACAGATATATTTGCCGTAGCCGTCTCG 55

*******************************************************

CP026245.1 CTCATCACGGGAAAGATCGTTTACATCTCGGACCAGGCTGCGACCATCTTGAACTGCAAG 900

Partial_per2 CTCATCACGGGAAAGATCGTTTACATCTCGGACCAGGCTGCGACCATCTTGAACTGCAAG 115

************************************************************

CP026245.1 CGGGAGGTGTTCAACAACGCCAATTTCGTGGAGTTTCTCACTCCTAAGGACGTCAGCGTG 960

Partial_per2 CGGGAGGTGTTCAACAACGCCAATTTCGTGGAGTTTCTCACTCCTAAGGACGTCAGCGTG 175

************************************************************

CP026245.1 TTCTACAGCTTCACCACGCCCTACCGGCTGCCCTCGTGGAGCATGTGCACCGGAGCAGAG 1020

Partial_per2 TTCTACAGCTTCACCACGCCCTACCGGCTGCCCTCGTGGAGCATGTGCACCGGAGCAGAG 235

************************************************************

CP026245.1 TCGTCTCCCACGGAGTGCATGCAGGAGAAGTCCTTCTTCTGCCGCATCAGTGGTGGGAAG 1080

Partial_per2 TCGTCTCCCACGGAGTGCATGCAGGAGAAGTCCTTCTTCTGCCGCATCAGTGGTGGGAAG 295

************************************************************

CP026245.1 GAGCGTGAAGGGGACCTCCAGTACTATCCTTTCCGTATGACTCCTTACCTCATGAAAGTC 1140

Partial_per2 GAGCGTGAAGGGGACCTCCAGTACTATCCTTTCCGTATGACTCCTTACCTCATGAAAGTC 355

************************************************************

CP026245.1 CAGGACGCTGAGCTGGCTGAGGAACAGTTCTGCTGCCTCCTGCTGGCTGAACGGGTTCAC 1200

Partial_per2 CAGGACGCTGAGCTGGCTGAGGAACAGTTCTGCTGCCTCCTGCTGGCTGAACGGGTTCAC 415

************************************************************

CP026245.1 TCCGGATACGAAGCTCCCAGAATCCCCCCTGACAAGCGCATCTTCACCACCACGCACACA 1260

Partial_per2 TCCGGATACGAAGCTCCCAGAATCCCCCCTGACAAGCGCATCTTCACCACCACGCACACA 475

************************************************************

CP026245.1 CCAAACTGTGTGTTCCAGGATGTGGACGAGAGGGCTGTTCCTCTGTTGGGTTACCTCCCT 1320

Partial_per2 CCAAACTGTGTGTTCCAGGATGTGGACGAGAGGGCTGTTCCTCTGTTGGGTTACCTCCCT 535

************************************************************

CP026245.1 CAGGACCTGATAGGGACCCCTCTGCTCCTCAAGCTGCACCCAAGTGACCGACCTTTAATG 1380

Partial_per2 CAGGACCTGATAGGGACCCCTCTGCTCCTCAAGCTGCACCCAAGTGACCGACCTTTAATG 595

************************************************************

CP026245.1 CTGGCCTTGCATCGCAAGATTCTGCAGTACGCCGGTCAGCCGTTCGACCACTCCTCGATC 1440

Partial_per2 CTGGCCTTGCATCGCAAGATTCTGCAGTACGCCGGTCAGCCGTTCGACCACTCCTCGATC 655

************************************************************

CP026245.1 CGCTTCTGTGCACGGAACGGCGAGTACGTCACCATAGACACCAGCTGGTCCAGCTTCGTC 1500

Partial_per2 CGCTTCTGTGCACGGAACGGCGAGTACGTCACCATAGACACCAGCTGGTCCAGCTTCGTC 715

************************************************************

CP026245.1 AACCCCTGGAGCCGCAAGGTCTCCTTCGTCATTGGCAGGCACAAAGTCCGCATGGGTCCT 1560

Partial_per2 AACCCCTGGAGCCGCAAGGTCTCCTTCGTCATTGGCAGGCACAAAGTCCGCATGGGTCCT 775

************************************************************

CP026245.1 GTGAACGAAGACGTTTTTGCAGTACCGGCTTTCCACGGAGGGAAGATCATGGACTCAGAC 1620

Partial_per2 GTGAACGAAGACGTTTTTGCAGTACCGGCTTTCCACGGAGGGAAGATCATGGACTCAGAC 835

************************************************************

CP026245.1 ATCCAGGAAATCAGTGAACAGATCCACAGGCTGCTGCTCCAACCGATCCACAACATGGGC 1680

Partial_per2 ATCCAGGAAATCAGTGAACAGATCCACAGGCTGCT------------------------- 870

***********************************

CP026245.1 TCCAGCGGTTACGGTAGCCACGGCAGCAACGGCTCCCATGAGCAACCCGTAAGCGTCGGC 1740

Partial_per2 ------------------------------------------------------------ 870

CP026245.1 TCATCCAGCGAGAGCAACGGGAACATGATGGTCACCGCCGCGGAGGAGCCAGACAAGGCC 1800

Partial_per2 ------------------------------------------------------------ 870

CP026245.1 AAGCCGCTCAGGACATTCCAGGAGATTTGCAAGGGCGTCCACATGCAGAAGAGCCAGGAC 1860

Partial_per2 ------------------------------------------------------------ 870

CP026245.1 TCCCAGGTCTCCCTGCAGTTGCCCTCGCTGCTGCCCCCCAGACCGGAGCTGAAGAAGTCC 1920

Partial_per2 ------------------------------------------------------------ 870

CP026245.1 ACTGACGCAGCTCAGAGGAGTCCAACGGTGCGTCTGAAGGACTCTGCGCCGCCTCTGCAA 1980

Partial_per2 ------------------------------------------------------------ 870

CP026245.1 GTCAGAGACGGTACCGCAGCCGTCGCGGATGACATCACCTGCAAGGACCAGGCCTCGTGC 2040

Partial_per2 ------------------------------------------------------------ 870

CP026245.1 TCCTACCAGCAGATCAGCTGCCTCGACAGCGTCATCAGGTACCTGGAGAGTTGTAACATC 2100

Partial_per2 ------------------------------------------------------------ 870

CP026245.1 CCCGTCACGGTGAAGAGGAAGTACCAGTTCTCCTCCAACACCACCTCCTCCAACTCTGAC 2160

Partial_per2 ------------------------------------------------------------ 870

CP026245.1 GACAGCAAGAAGGGCTCAGAGGATGGCATGCATGTGTGTCAGGATACTACCACAGATCCT 2220

Partial_per2 ------------------------------------------------------------ 870

CP026245.1 TTGATGCTCAGCACCCAGCCAGGCCTGTCGAACATGAAAGCACCTAAAAAGCCTCTGGCC 2280

Partial_per2 ------------------------------------------------------------ 870

CP026245.1 CCCCTGACTCTGCCCAGCAAGGCTGAGAGTGTGGTGTCCATCACTTCCCAGTGCAGCTAC 2340

Partial_per2 ------------------------------------------------------------ 870

CP026245.1 AGCAGCACCATTGTCCACGTGGGAGACAAGAAGCCTCAGCCCGAGTCCGAGATAATCGGG 2400

Partial_per2 ------------------------------------------------------------ 870

CP026245.1 GACGTGGCGGAGAGCCCAGCTCCTCCGGCCCTGCCCGTCAGCACGGTGTCTCCGCCCAGC 2460

Partial_per2 ------------------------------------------------------------ 870

CP026245.1 CAGGAGAAGGAGGCCTACAAAAGGCTGGGACTAACCAAACAGGTGCTGGCGGCGCACACC 2520

Partial_per2 ------------------------------------------------------------ 870

CP026245.1 CAGAAAGAGGAGCAGGTCTTCCTCAACCGCTGCCGAGAACTGCGCCACGCCAGGAGCGTC 2580

Partial_per2 ------------------------------------------------------------ 870

CP026245.1 CAGAAGGACTGTTCCAAATACTTGCACAGGCAGAAGGATCCAGCCAACGCTAAAGAATCC 2640

Partial_per2 ------------------------------------------------------------ 870

CP026245.1 TCTGGACCTCGAGGTGCCACCAAACAGGGCCCCGCCCGACTGGAGACTGCCGCCAAGAAG 2700

Partial_per2 ------------------------------------------------------------ 870

CP026245.1 GGCAACCGCAGCAAGAAGTCCAAGAAATCTCGCATGAAGCACCCCGACTCGTCCGACAGC 2760

Partial_per2 ------------------------------------------------------------ 870

CP026245.1 GCTGTGTCCAACCGCAAACCCCGCCCCCCGCTCCAGGGTCTCAACCAGACCTCGTGGTCG 2820

Partial_per2 ------------------------------------------------------------ 870

CP026245.1 CCGTCGGAAGCCTCGCAGTCTGCGTTTAGCGCCTCCTACCCTGCCATGGTGCCTGCATAC 2880

Partial_per2 ------------------------------------------------------------ 870

CP026245.1 CCGCTCTACCCCCCAGCCCCTGCTGCCCCCGCTCAGGCCCCTAACCCCTCCCTTTCTACA 2940

Partial_per2 ------------------------------------------------------------ 870

CP026245.1 GGCTTCGGAGAAGGGCAGAGCGCCCAAGCCCCACCTACCGCCACTCCATTCCCCACCCCA 3000

Partial_per2 ------------------------------------------------------------ 870

CP026245.1 ATCGTTACGCCGGTGGTGGCTCTGGTGCTGCCAAATTACATTTTCCCCCAAATGGGACAG 3060

Partial_per2 ------------------------------------------------------------ 870

CP026245.1 CTTGGGCCGATGGGGCAGCTGGGGGCTGCTCCTGTTCCAGCGTTTTTTCTCGAGCAGACA 3120

Partial_per2 ------------------------------------------------------------ 870

CP026245.1 CAGGCACAACCTGCGTTTGCTACCCAGCAGCCCTTTCAGCCCCCGCAGCCGGCCTTCACC 3180

Partial_per2 ------------------------------------------------------------ 870

CP026245.1 ATGCAAACACAACCCCCCTTCACCAACCAACAGCCGTTCCCTCCCTTCGCCGCCCAGCCG 3240

Partial_per2 ------------------------------------------------------------ 870

CP026245.1 TCTTTCCCCATACAGACACAGTTTGTCGCTCAATCCCCTTTCCCTACGCAGCCCTTCCCC 3300

Partial_per2 ------------------------------------------------------------ 870

CP026245.1 TTCAGCCTGGCCCCGGAGCCCCCTAAAGCCCTGGTCATGGAGTCTCGAGAAGGGGCGGCG 3360

Partial_per2 ------------------------------------------------------------ 870

CP026245.1 TCGCGCTCCTCCACCCCGGTCTACGGGACGCGGGAGCCAGCAGCGTCGCCGCCGCTGTTC 3420

Partial_per2 ------------------------------------------------------------ 870

CP026245.1 GAGTCGAGATGCAGCTCGCCGCTGCATCTCAATCTGCTGAGCATGGAGGAGGGTCAGCGG 3480

Partial_per2 ------------------------------------------------------------ 870

CP026245.1 TCGATGGAACGTCAGGACATCACGGCGCCCTCTGCTGGAGGTCAGGGCAGCACCTCAGCA 3540

Partial_per2 ------------------------------------------------------------ 870

CP026245.1 GCGGCGTCGGGAGTCGAGGAGCGGAACGGTGGCTCAGTCAAGACTGAAAACCACCAACAG 3600

Partial_per2 ------------------------------------------------------------ 870

CP026245.1 GTGGAGTGTCTCGGAGACGGCGCTCACAGTGACGGTAACTCCTCATCCTGCGACCTGCTG 3660

Partial_per2 ------------------------------------------------------------ 870

CP026245.1 GACATCCTTCTGCAGGAGGACTCCCACTCCGGCACTGGATCAGCCACCTCTGGGTCCATG 3720

Partial_per2 ------------------------------------------------------------ 870

CP026245.1 GGCTCGAGATCAGGCTCTGGATCGGGCTCAGGATCGGGCTCAGGATCGGGCTCTAGATCA 3780

Partial_per2 ------------------------------------------------------------ 870

CP026245.1 GGATCGGGCTCGGGCTGTAACGGCTGTGGTACCTCCGGGAGCGGAGGCTCCGGCAGCAGA 3840

Partial_per2 ------------------------------------------------------------ 870

CP026245.1 ACAGGAAGCAGCAACACCAGCAAATACTTTGGCAGCATCGACTCCCTGGAACACGACCCA 3900

Partial_per2 ------------------------------------------------------------ 870

CP026245.1 AAGGGGAAGGTCAAACCCAAGACGAGGAGCAAAGGAGGCTCTAACGGTGGCCAGCCGCAG 3960

Partial_per2 ------------------------------------------------------------ 870

CP026245.1 GCCAAGGTCTCGAGTCCAGGGGAGGAGGAGCACTTCAACAAATACGTTCTCCAGGAGCCA 4020

Partial_per2 ------------------------------------------------------------ 870

CP026245.1 CTGTGGCTGCTGATGGCCAATGCTGATGACAAGGTCATGATGACATATCAGTTACCCACC 4080

Partial_per2 ------------------------------------------------------------ 870

CP026245.1 AGGGACATTCAGAAGGTTCTGCGGGAAGACAAGGAGCGACTGAGGCAGATGCGGAAGAGT 4140

Partial_per2 ------------------------------------------------------------ 870

CP026245.1 CAGCCTCACTTCTCCTCAGACCAGCGACGGGAGCTCCTGGACGAACACCCCTGGATGAGG 4200

Partial_per2 ------------------------------------------------------------ 870

CP026245.1 AGGGGAGGTCTGCCCACTGCCGTCAATGTGAAGGAATGTATGTACTGCGAGGACGCAGCA 4260

Partial_per2 ------------------------------------------------------------ 870

CP026245.1 GGGCCCATCCAGGAAGACCTGTCGCACATGGACATGGGCGAGCAGCTGGGCGAGGGGCTG 4320

Partial_per2 ------------------------------------------------------------ 870

CP026245.1 AGGCCCGACGGACATAACAGCCAATCAAAGGAGTCGCAGCCCCGACCCGACACTGGCTCA 4380

Partial_per2 ------------------------------------------------------------ 870

CP026245.1 TGA 4383

Partial_per2 --- 870

**Cry1**

CP026253.1 ATGGCCCGAAATTCCATCCACTGGTTCCGAAAGGGCCTCCGTCTCCACGACAACCCCGCG 60

Partial_cry1 ------------------------------------------------------------ 0

CP026253.1 CTAAAGGAGGCAGTCGTGGGAGCGGACACGGTGCGCTGCGTTTACTTCCTGGACCCATGG 120

Partial_cry1 ------------------------------------------------------------ 0

CP026253.1 TTCGCAGGCTCCTCCAATGTCGGAGTCAACAGGTGGAGGTTTCTCCTCCAGTGTTTGGAG 180

Partial_cry1 ------------------------------------------------------------ 0

CP026253.1 GATCTGGACGCCAGCCTGCGAAAGCTCAACTCCCGCCTTTTTGTCATCAGGGGCCAACCA 240

Partial_cry1 ------------------------------------------------------------ 0

CP026253.1 GCCAACGTGTTCCCGCGGCTCTTTAAGGAGTGGAAGATCTCCCGGCTCACCTTTGAGTAC 300

Partial_cry1 ------------------------------------------------------------ 0

CP026253.1 GATTCAGAGCCTTTTGGTAAGGAGAGAGACGCCGCCATCAAGAAGCTGGCCATGGAGGCC 360

Partial_cry1 ------------------------------------------------------------ 0

CP026253.1 GGCGTGGAGGTCAACGTCAAGATATCACACACCCTCTACGACCTGGACAAGATCATTGAG 420

Partial_cry1 ------------------------------------------------------------ 0

CP026253.1 CTGAATGGGGGGCAGCCTCCTCTCACCTACAAGCGTTTCCAGACCCTGATCAGTCGACTG 480

Partial_cry1 ----------GGCAGCCTCCTCTCACCTACAAGCGTTTCCAGACCCTGATCAGTCGACTG 50

**************************************************

CP026253.1 GATCCTCCTGAGCTGCCAGCAGACGCCTTGTCGGACACCCTGATGGGACGCTGTGTCACC 540

Partial_cry1 GATCCTCCTGAGCTGCCAGCAGACGCCTTGTCGGACACCCTGATGGGACGCTGTGTCACC 110

************************************************************

CP026253.1 CCAATCTCCGACGACCACGGCGACAAGTACGGGGTCCCATCGTTAGAGGAGCTAGGCTTT 600

Partial_cry1 CCAATCTCCGACGAACACGGCGACAAGTACGGGGTCCCATCGTTAGAGGAGCTAGGCTTT 170

************** *********************************************

CP026253.1 GACATAGAGGGCCTGCCTTCAGCAGTGTGGCCGGGAGGAGAGACTGAGGCTCTGACCAGG 660

Partial_cry1 GACATAGAGGGCCTGCCTTCAGCAGTGTGGCCGGGAGGAGAGACTGAGGCTCTGACCAGG 230

************************************************************

CP026253.1 ATCGAGCGCCACCTGGAGAGAAAAGCGTGGGTGGCTAATTTTGAGCGCCCCAGGATGAAC 720

Partial_cry1 ATCGAGCGCCACCTGGAGAGAAAAGCGTGGGTGGCTAATTTTGAGCGCCCCAGGATGAAC 290

************************************************************

CP026253.1 GCCAACTCGCTGCTGGCCAGCCCAACAGGCCTCAGCCCCTACCTGCGCTTTGGCTGCCTC 780

Partial_cry1 GCCAACTCGCTGCTGGCCAGCCCAACAGGCCTCAGCCCCTACCTGCGCTTTGGCTGCCTC 350

************************************************************

CP026253.1 TCCTGCCGCCTTTTCTACTTCAAGCTCACAGACCTGTACCGCAAGGTGAAGAAGAACAAC 840

Partial_cry1 TCCTGCCGCCTTTTCTACTTCAAGCTCACAGACCTGTACCGCAAGGTGAAGAAGAACAAC 410

************************************************************

CP026253.1 TCCCCTCCGCTTTCTCTTTATGGCCAGTTACTGTGGCGCGAGTTCTTCTACACGGCGGCA 900

Partial_cry1 TCCCCTCCGCTTTCTCTTTATGGCCAGTTACTGTGGCGCGAGTTCTTCTACACGGCGGCA 470

************************************************************

CP026253.1 ACCAACAACCCGCGCTTTGACAAGATGGAGGGTAACCCCATCTGCGTCCGCATCCCCTGG 960

Partial_cry1 ACCAACAACCCGCGCTTTGACAAGATGGAGGGTAACCCCATCTGCGTCCGCATCCCCTGG 530

************************************************************

CP026253.1 GACCGAAATCCAGAGGCTCTCGCCAAGTGGGCTGAGGCCAAGACGGGCTTTCCCTGGATA 1020

Partial_cry1 GACCGAAATCCAGAGGCTCTCGCCAAGTGGGCTGAGGCCAAGACGGGCTTTCCCTGGATA 590

************************************************************

CP026253.1 GACGCCATCATGACTCAGCTGAGGCAGGAGGGCTGGATCCATCACCTGGCCAGGCACGCG 1080

Partial_cry1 GACGCCATCATGACTCAGCTGAGGCAGGAGGGCTGGATCCATCACCTGGCCAGGCACGCG 650

************************************************************

CP026253.1 GTGGCCTGCTTCCTCACCAGGGGGGACCTGTGGATCAGCTGGGAGGAAGGGATGAAGGTC 1140

Partial_cry1 GTGGCCTGCTTCCTCACCAGGGGGGACCTGTGGATCAGCTGGGAGGAAGGGATGAAGGTC 710

************************************************************

CP026253.1 TTTGAGGAGTTGCTTCTCGATGCGGACTGGAGTGTGAACGCCGGCAGCTGGATGTGGCTG 1200

Partial_cry1 TTTGAGGAGTTGCTTCTCGATGCGGACTGGAGTGTGAACGCCGGCAGCTGGATGTGGCTG 770

************************************************************

CP026253.1 TCCTGCAGCTCATTTTTCCAGCAGTTTTTCCACTGCTACTGTCCTATGGGCTTCGGCCGG 1260

Partial_cry1 TCCTGCAGCTCATTTTTCCAGCAGTTTTTCCACTGCTACTGCCCTATGGGCTTCGGCCGG 830

***************************************** ******************

CP026253.1 CGCACCGACCCCAACGGGGACTTCATCAGACGATACCTACCTCTCCTCCGAGGTTTCCCC 1320

Partial_cry1 CGCACCGACCCCAACGGGGACTTCATCAGACGATACCTACCTCTCCTCCGAGGTTTCCCC 890

************************************************************

CP026253.1 GCCAAATACATCTACGACCCGTGGAACGCTCCGGAGTCCGTGCAGGCGGCCGCCAAGTGC 1380

Partial_cry1 GCCAAATACATCTACGACCCGTGGAACGCTCCGGAGTCCGTGCAGGCGGCCGCCAAGTGC 950

************************************************************

CP026253.1 ATAATTGGCGTCCATTACCCGAAGCCCATGGTGCATCACGCGGAGGCAAGCCGACTCAAC 1440

Partial_cry1 ATAATTGGCGTCCA---------------------------------------------- 964

**************

CP026253.1 ATCGAGAGGATGAAGCAGATCTACCAGCAACTTAGCCGATACAGGGGACTAGGCCTGCTG 1500

Partial_cry1 ------------------------------------------------------------ 964

CP026253.1 GCATCAGTGCCGTCCACAAATGGTACCGGTAATGGAGGAATGATGGCCTACTCACCCGAG 1560

Partial_cry1 ------------------------------------------------------------ 964

CP026253.1 GGGCAGCAGCTAGGGACCAACAACAACAACTCACATTTGCCTGCAGTTTCTGGGAGCTCC 1620

Partial_cry1 ------------------------------------------------------------ 964

CP026253.1 GTTGCAACAGGAAGCGGCAGCGGGAGCGTCCTACTCAACTTTGACAGTGAAGAACAGACA 1680

Partial_cry1 ------------------------------------------------------------ 964

CP026253.1 CGGCCTAGCAGTGTCGGACAACAGCAACAGCAGCAACATCATCAACAACCTCAACAACAT 1740

Partial_cry1 ------------------------------------------------------------ 964

CP026253.1 CAGCAGCAACATCAACAACAGCAGCAACATCAACAACATCATCAACAGCAGCCGCAGCAG 1800

Partial_cry1 ------------------------------------------------------------ 964

CP026253.1 CATGGATACCACTCAGTGCCAGACGCCAGACAGACCATCACCAGCAGCCAACTCTTCCAC 1860

Partial_cry1 ------------------------------------------------------------ 964

CP026253.1 GAGTTCGCTGTGCCTCAACACCCTGGACTCTTCCTTCACGGCAGAAGCTGCGTCACAGGA 1920

Partial_cry1 ------------------------------------------------------------ 964

CP026253.1 AAGCGGGAGAGGGAGTCGGAACGCGAAGGGTTGGGGGAGGAAGACCCGGCGTCCTACTCC 1980

Partial_cry1 ------------------------------------------------------------ 964

CP026253.1 GTGCACAAGATGCAGAGGCAAAGTGCAGAGGTGAGCCAGCGTGCCATCGCACCACACGGT 2040

Partial_cry1 ------------------------------------------------------------ 964

CP026253.1 GATGAAGTGAAGTAG 2055

Partial_cry1 --------------- 964
